# An examination of the mechanisms driving the therapeutic effects of an AAV expressing a soluble variant of VEGF receptor-1

**DOI:** 10.1371/journal.pone.0305466

**Published:** 2024-07-11

**Authors:** Seo Yun Moon, Hee Jong Kim, Jin Kwon Kim, Jin Kim, Jun-Sub Choi, So-Yoon Won, Keerang Park, Steven Hyun Seung Lee

**Affiliations:** 1 Institute of New Drug Development Research, CdmoGen Co., Ltd., Seoul, Korea; 2 CdmoGen Co., Ltd., Cheongju, Korea; Fudan University, CHINA

## Abstract

In previous animal model studies, we demonstrated the potential of rAAV2-sVEGFRv-1, which encodes a truncated variant of the alternatively spliced soluble version of VEGF receptor-1 (VEGFR1), as a human gene therapy for age-related macular degeneration (AMD) and diabetic retinopathy (DR). Here, we elucidate in vitro some of the mechanisms by which rAAV2-sVEGFRv-1 exerts its therapeutic effects. Human umbilical vein endothelial cells (HUVECs) were infected with rAAV2-sVEGFRv-1 or a control virus vector in the presence of members of the VEGF family to identify potential binding partners via ELISA, which showed that VEGF-A, VEGF-B, and placental growth factor (PlGF) are all ligands of its transgene product. In order to determine the effects of rAAV2-sVEGFRv-1 on cell proliferation and permeability, processes that are important to the progression AMD and DR, HUVECs were infected with the therapeutic virus vector under the stimulation of VEGF-A, the major driver of the neovascularization that characterizes the forms of these conditions most associated with vision loss. rAAV2-sVEGFRv-1 treatment, as a result, markedly reduced the extent to which these processes occurred, with the latter determined by measuring zonula occludens 1 expression. Finally, the human microglial HMC3 cell line was used to show the effects of the therapeutic virus vector upon inflammatory processes, another major contributor to angiogenic eye disease pathophysiology, with rAAV2-sVEGFRv-1 reducing therein the secretion of pro-inflammatory cytokines interleukin (IL)-1β and IL-6. Combined with our previously published in vivo data, the in vitro activity of the expressed transgene here further demonstrates the great promise of rAAV2-sVEGFRv-1 as a potential human gene therapeutic for addressing angiogenic ocular conditions.

## Introduction

Among diseases of the eye, age-related macular degeneration (AMD) and diabetic retinopathy (DR) are two of the leading causes of blindness worldwide [1, 2], with neovascularization (NV) being the process most associated with the development of vision loss [3]. Both AMD and DR are degenerative disorders [3, 4], with the former potentially progressing to either the dry subtype of the disease, whose advanced form is noted for the presence of geographic atrophy, or the wet subtype, also known as neovascular AMD (nvAMD) [3], in which NV originating from the choroid and driven by vascular endothelial growth factor (VEGF) occurs. VEGF is also responsible for the development of retinal NV upon the progression of DR, a complication of diabetes mellitus (DM), from its non-proliferative form to proliferative diabetic retinopathy (PDR) [4]. Besides the role it plays in the vascular aspects of AMD and DR, VEGF is also implicated in additional processes [5, 6] involved in the pathophysiology of these multifactorial disorders [7, 8], including inflammation [2, 5, 9] and vessel permeability [5, 10]. Given the influential effects of VEGF, it is unsurprising that anti-VEGF therapeutics represent the current standard of care for nvAMD and DR.

The most widely-used anti-VEGF drugs include bevacizumab, a monoclonal antibody used off-label due to the economic benefits it offers [11]; ranibizumab, a fab fragment based on bevacizumab; aflibercept, a recombinant fusion protein; and brolucizumab, a single-chain antibody fragment [5, 11]. Administered via intravitreal injection, these protein-based drugs feature relatively short durations of action, though advances have been made with each successively approved therapeutic. Despite these improvements and alternative treatment schedules that maximize the intervals between injections [11], multiple administrations are necessary per year for DR and AMD patients. Being economically and procedurally burdensome, patient compliance suffers [11, 12], leading to less favorable outcomes. Given this disadvantage of current therapeutics and that one quarter [2] of the 592 million diabetes DM estimated by 2035 [13] are projected to develop sight-threatening DR while AMD is expected to affect 288 million patients by 2040 [1], gene therapy may provide an ideal solution to this looming global health crisis. The use of a recombinant adeno-associated virus (rAAV), which has a long-established research history as a gene therapy delivery vector for ocular conditions [6, 14], may then provide lasting therapeutic effects, as rAAVs transduce dividing and non-dividing cells to elicit long-term transgene expression while being non-pathogenic [15]. In fact, the approval of voretigene neparvovec-rzyl (Luxturna; Spark Therapeutics, Philadelphia, PA) in 2017 thus demonstrated the safety and efficacy of serotype 2 rAAVs for these kinds of applications [16], with additional research in the field of rAAV-delivered gene therapeutics continuing to this day [17].

In mammals, the VEGF family consists of five members, VEGF-A, VEGF-B, VEGF-C, VEGF-D, and placental growth factor (PlGF), which interact with three numbered receptor tyrosine kinases [18]. VEGF-C, VEGF-D, and VEGF receptor-3 are mainly involved in lymphangiogenesis [19] and lie beyond the scope of this manuscript. Meanwhile, the interactions of VEGF-A, the most studied member of the family, with VEGF receptor-2 serve as the major driver and master regulator of angiogenesis [20], making VEGF-A an attractive therapeutic target. Indeed, bevacizumab, ranibizumab, and brolucizumab all target VEGF-A to exert their anti-VEGF effects [5]. Meanwhile, VEGF receptor-1 (VEGFR1), also called Flt-1, binds VEGF-B and PlGF in addition to VEGF-A [6]. Given the growing recognition of the roles played by VEGFR1 signaling [5] and PlGF [21] in pathological processes associated with angiogenic ocular conditions, targeting VEGFR1 activity may present an opportunity for the development of a human gene therapy for AMD and DR.

As such, we have developed and explored the therapeutic potential [22, 23] of a rAAV encoding a truncated variant of sFlt-1, an alternatively-spliced soluble version of full-length VEGFR1 that binds VEGF-A with a high affinity [24]. The resulting virus vector, rAAV2-sVEGFRv-1, was shown to be anti-angiogenic in a mouse model of laser-induced choroidal neovascularization (CNV), a nvAMD model system, by reducing the extent to which CNV occurred. It also protected against the infiltration of inflammatory cells into CNV lesions, macrophages and leukocytes in particular, while having anti-apoptotic and anti-fibrotic effects as well [22]. Having demonstrated that rAAV2-sVEGFRv-1 curtails the VEGF-driven neovascular activity that characterizes PDR [4], additional therapeutic effects were explored in a streptozotocin (STZ)-induced diabetic mouse model portraying the earlier stages of DR [23]. In the study, rAAV2-sVEGFRv-1 protected against retinal pericyte loss, an important early contributor to DR pathophysiology that helps drive disease progression [4] and the development of leaky vessels. Indeed, rAAV2-sVEGFRv-1 reduced retinal vascular permeability, acellular capillary development, and the thinning of cell layers in the inner retina, suggesting that the therapeutic virus vector may be neuroprotective as well [23]. Having thus demonstrated the in vivo efficacy of rAAV2-sVEGFRv-1, it became necessary to elucidate the mechanism by which it works to proceed in its development as a potential human gene therapeutic.

Here, we examined the mechanistic activity of rAAV2-sVEGFRv-1 in vitro using human umbilical vein endothelial cells (HUVECs) to demonstrate that the virus vector is able to effectively express its transgene, and the resulting sFlt-1 variant was shown to interact with VEGF-A, VEGF-B, and PlGF, the natural binding partners of VEGFR1. Via these interactions, rAAV2-sVEGFRv-1 treatment was able to reduce endothelial cell proliferation and tight junction structural dysfunction despite VEGF-A stimulation, which are respectively associated with the pathological processes of angiogenesis and vascular permeability. The anti-inflammatory effects of rAAV2-sVEGFRv-1 were shown in human microglial HMC3 cells, where it reduced the secretion of the pro-inflammatory cytokines IL-1β and IL-6. Taken together, these results demonstrate the mechanistic bases underlying the great promise exhibited by rAAV2-sVEGFRv-1 as a potential gene therapy for AMD and DR.

## Materials and methods

### Virus vector preparation

rAAV2-sVEGFRv-1 and rAAV2-GFP, used here as a negative control, were generated as previously described [22]. For the former, a section of the human VEGF receptor-1 gene mRNA (XM_017020485.1; NCBI Reference Sequence, NIH) spanning nucleotide positions 282 to 2,253 was inserted into a pAAV-F.IX cis plasmid containing a SV40 polyadenylation signal and both ITRs, with the transgene under the control of a CMV promoter, to yield the recombinant therapeutic virus vector. rAAV2-GFP was produced by inserting an EGFP expression cassette in an analogous manner. All virus vectors used in this study were supplied by CdmoGen Co., Ltd. (Cheongju, Korea).

### Cell culture and infection

HUVECs (C2517A; Lonza, Basel, Switzerland) were cultured in EBM-2 Endothelial Cell Growth Basal Medium (CC-3156; Lonza) supplemented with EGM-2 Endothelial SingleQuots Kit (CC-4176; Lonza) and experimentation performed in dishes coated with 0.1% gelatin (LS023-01; Welgene, Inc., Gyeongsan, Korea). HMC3 cells (CRL-3304; ATCC, Manassas, VA) were cultured in Minimum Essential Medium Eagle with 1 mM sodium pyruvate and 1.5 mg/mL sodium bicarbonate (LM007-54; Welgene) supplemented with 10% fetal bovine serum (FBS) and 1% penicillin-streptomycin. To induce starvation, HUVECs were incubated in EBM-2 medium supplemented with EGM-2 but without adding VEGF-A and FBS prior to VEGF-A treatment, whereas HMC3 cells were incubated in serum-free medium before being treated with PlGF. All cells were maintained at 37°C under a humidified 5% CO_2_ atmosphere. Unless otherwise specified, cell infections were performed by the respective virus vector at 25,000 MOI.

### RT-PCR

TRIzol reagent (Thermo Fisher Scientific, Waltham, MA) was used according to the manufacturer’s instructions to extract total RNA from the infected HUVECs, with the total RNA being treated with DNase I (Thermo Fisher Scientific) to prevent contamination from viral genomic DNA. Reverse Transcription Master mix (EBT-1542; Elpis-Biotech, Daejeon, Korea) was used to synthesize a cDNA using the primer as follows, 5’-AGTGCAGGGTCCGAGGTATCTTGCCGGCCTCGAGCTA-3’, whereas PCR was performed using the following primers for sVEGFRv-1 (forward, 5’-CGTGTAAGGAGTGGACCATC-3’; reverse, 5’-TAAGACCGCTTGCCAGCTAC-3’) and β-actin (forward, 5’-TGAAGATCAAGATCATTGCTC-3’; reverse, 5’-TGCTTGCTGATCCACATCTG-3’).

### ELISA

HUVECs were seeded in 6-well plates at a density of 1 x 10^5^ cells per well and infected with rAAV2-sVEGFRv-1. 72 hours later, infected HUVECs were reseeded into 12-well plates at a density of 1 x 10^4^ cells per well and cultured for an additional 24 hours, 3 days, or 5 days, after which the supernatant was harvested. Quantification of the amount of sVEGFRv-1 transgene product expressed was performed using a Human VEGFR1/Flt-1 Quantikine ELISA Kit (SVR100C; R&D Systems, Minneapolis, MN) according to the manufacturer’s protocol.

The binding assay to determine the levels to which sVEGFRv-1 interacted with the known ligands of VEGFR1 was performed by seeding, infecting, and reseeding HUVECs as described above for ELISA. After culturing the cells for an additional 24 hours, the supernatant was harvested and assayed with the Human VEGFR1/Flt-1 Quantikine ELISA Kit in the presence of the natural binding partners of VEGFR1. Briefly, the 96-well plates were coated with 200ng of either VEGF-A (293-VE-050/CF), VEGF- B (751-VEB-025), or PlGF (264-PGB-050/CF), all of which were also obtained from R&D Systems, and incubated overnight at 4°C before being blocked with 2% bovine serum albumin (BSA) in PBS for 2 hours at room temperature and the assay performed in accordance with the manufacturer’s instructions.

### Determination of LDH cytotoxicity and cell viability

To determine whether rAAV2-sVEGFRv-1 is cytotoxic, HUVECs were seeded in 96-well plates at a density of 1 x 10^4^ cells per well and treated with rAAV2-sVEGFRv-1 for either 24 hours or 7 days prior to the use of the CyQUANT LDH Cytotoxicity Assay (C20301; Invitrogen, Waltham, MA) in accordance with the manufacturer’s protocol. LHD activity in impaired cells was then determined by subtracting the background absorbance taken at 680 nm from the measured absorbance at 490 nm before calculating the cytotoxicity percentage using the following formula: % cytotoxicity = [(compound-treated LDH activity–spontaneous LDH activity) / (maximum LHD activity–spontaneous LHD activity)] x 100. The cell suspension was then mixed with Trypan Blue (1450021; Bio-Rad Laboratories, Hercules, CA) in a 1:1 ratio and incubated for 1–2 minutes at room temperature before cell viability was assessed using a LUNA-FL Dual Fluorescence Cell Counter (Logos Biosystems, Anyang, Korea).

### Proliferation assay

To determine the effects rAAV2-sVEGFRv-1 has upon VEGF-A-induced endothelial cell proliferation, a Cell Counting Kit-8 (CCK-8) assay was performed on HUVECs seeded into 96-well plates with EBM-2 and EGM-2 at a density of 4 x 10^3^ cells per well. The cells were pretreated with rAAV2-sVEGFRv-1 for 3 days before being stimulated with 30 ng/mL of VEGF-A protein, with the cells also having been subjected to 24 hours of starvation leading up to VEGF-A stimulation. A mixture of 30 μL of CCK-8 in 300 μL of starvation medium, the composition of which was described above alongside other cell culture media information, was then added to each well and the samples incubated for an additional 2 hours before absorbance was measured at 450 nm using a BioTek Epoch Microplate Spectrophotometer (Agilent Technologies, Santa Clara, CA).

### Western blot analysis

Total proteins were extracted from samples using an extraction buffer containing 50 mM Tris (pH 7.4), 150 mM NaCl, 1% Triton X-100, 1% sodium deoxycholate, 0.1% SDS, and 1 mM PMSF. After vortexing for 30 minutes followed by centrifugation at 12,000 rpm for 15 minutes, the supernatants were collected and protein concentrations determined by the BCA method. 30 μg of the protein samples were heated at 95°C for 5 minutes, resolved on a 10% SDS-PAGE gel, and transferred onto a polyvinylidene difluoride (PVDF) membrane, which was blocked with 5% skim milk in Tris-buffered saline containing 0.1% Tween-20 for 1 h and then incubated overnight with the primary antibody at 4°C. Antibodies used include those for phospho-PKD/PKCμ (Ser744/748) (2054S; Cell Signaling Technology, Danvers, MA), PKC mu/PKD (ab108963; Abcam, Cambridge, UK), GAPDH (sc-166574; Santa Cruz Biotechnology, Dallas, TX), ZO-1 (33–9100; Invitrogen), IL-6 (MBS9606748; MyBioSource, San Diego, CA), and IL-1β (ab254360; Abcam). After washing three times with TBST for 10 minutes each, the membrane was incubated with goat anti-mouse HRP (A0216; Beyotime Biotechnology, Nantong, China) or goat anti-rabbit HRP (A0208; Beyotime Biotechnology) secondary antibody at a 1:1000 ratio for 2 hours at room temperature and the proteins visualized via a chemiluminescent substrate (SuperSignal Ultra; Pierce Chemical, Rockford, IL) using a LuminoGraph II chemiluminescence imaging system (WSE-6200; Atto Corporation, Tokyo, Japan).

### Immunocytochemistry

HUVECs were seeded into 2-well chambered slides (80296; ibidi, Gräfelfing, Germany) at a density of 1.5 x 10^5^ cells per well, whereas HMC3 cells were seeded into 4-well chambered slides (154526PK, Thermo Fisher Scientific) at a density of 5 x 10^3^ cells per well. After being infected with rAAV2-sVEGFRv-1 or the control virus vector for 48 hours, the cells were starved for 24 hours and treated with either 30 ng/mL of VEGF-A or 300 ng/mL of PlGF for 1 or 4 hours, respectively. The cells were then fixed with 4% paraformaldehyde and permeabilized using 0.2% Triton X-100 and 0.5% BSA for 15 minutes for HUVECs and 40 minutes for HMC3 cells. After washing with PBS, HUVECs underwent an additional step wherein they were treated with 5% normal goat serum blocking solution (S-1000-20; Vector Laboratories, Newark, CA) in PBST for 30 minutes. Following overnight incubation at 4°C with the appropriate primary antibody, including those for ZO-1, IL-1β, IL-6, or Iba1 (ab5076; Abcam), the cells were washed with 0.5% BSA in PBS before being incubated at room temperature for 2 hours with the secondary antibodies Alexa Fluor 488 or 594 (Thermo Fisher Scientific) and then counterstained with DAPI (D9542; Sigma-Aldrich, St. Louis, MO) for nuclear visualization purposes. Fluorescence microscopy was then performed, with images captured using a Ts2-FL microscope (Nikon, Tokyo, Japan).

### Image and statistical analysis

To quantify immunoblot band densities and the intensities of immunofluorescence signals, image analysis was performed using ImageJ software (National Institutes of Health, Bethesda, MD). GraphPad Prism software (version 8.0; GraphPad Software, Boston, MA) was used to analyze generated data. Statistical significance was assessed using an unpaired two-tailed Student’s t-test or an ANOVA with Student-Newman-Keuls post hoc analysis. Quantitative data are presented as means and the standard error of the mean, and differences were considered significant at p < 0.001 or p < 0.05. All image files are available from the Harvard Dataverse database (https://doi.org/10.7910/DVN/LSWMHT).

## Results

### In vitro characterization of rAAV2-sVEGFRv-1

**Fig 1:**
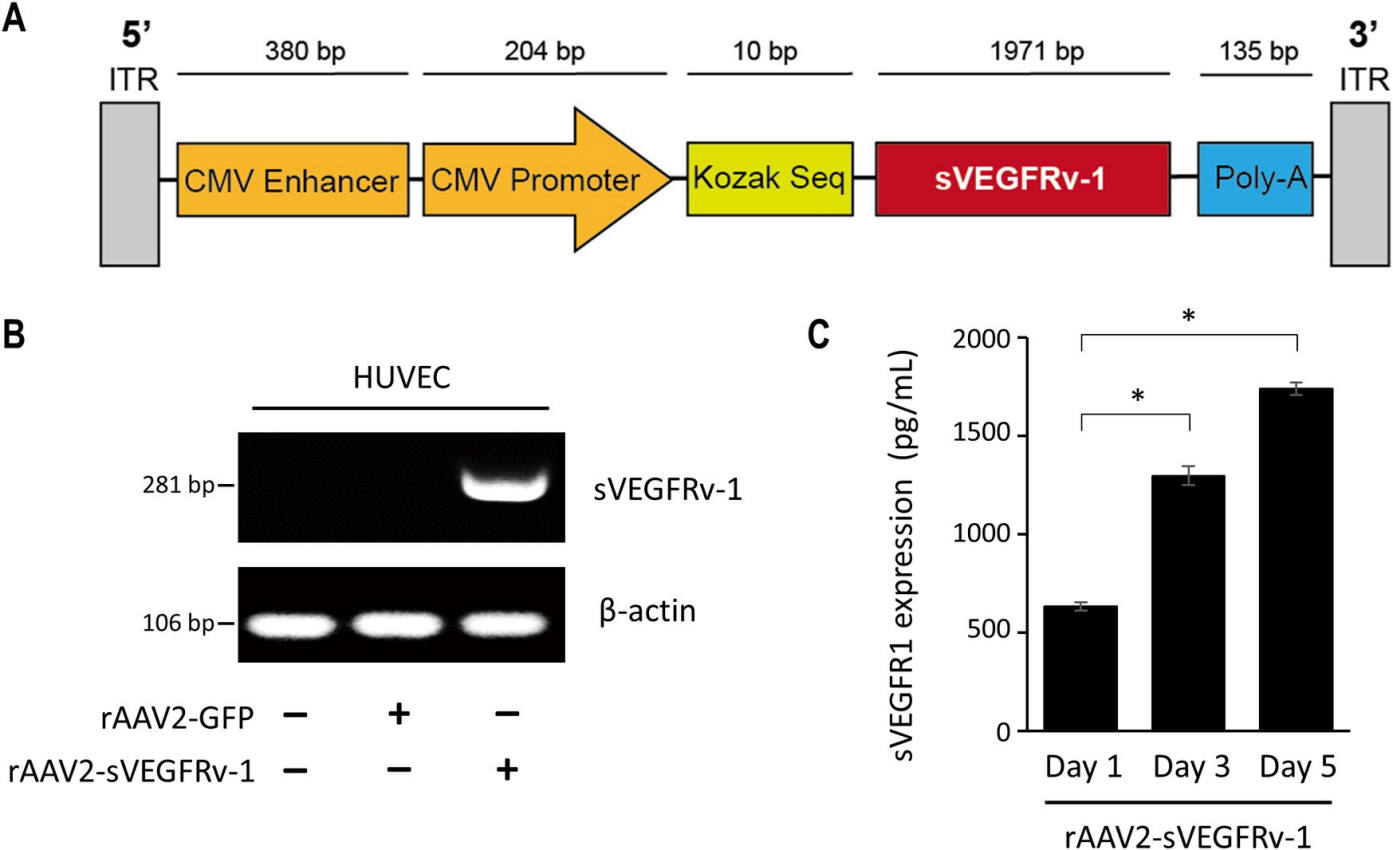
rAAV2-sVEGFRv-1 transgene expression. (a) Schematic of rAAV2-sVEGFRv-1, which was able to infect HUVECs to express a transgene product that was identified via RT-PCR (b) and ELISA (c) as sVEGFRv-1, with protein levels increasing with time. Data represented as mean ± SEM (n = 3). * p < 0.001.

Upon infecting HUVECs with rAAV2-sVEGFRv-1 (Fig 1A) at 25,000 MOI, the ability of the therapeutic virus vector to successfully express its transgene was determined in vitro. RT-PCR showed that rAAV2-sVEGFRv-1 treatment specifically increased sVEGFRv-1 mRNA levels (Fig 1B), leading to sVEGFRv-1 protein expression, measured via ELISA (Fig 1C). Protein levels continued to rise as transgene expression progressed, from 633.5 ± 22.00 pg/mL 1 day following 72 hours of rAAV2-sVEGFRv-1 infection to 1298.0 ± 47.57 pg/mL and 1739.9 ± 32.71 pg/mL at 3 days and 5 days after, respectively, demonstrating the efficacy of the recombinant adeno-associated virus vector as a gene therapy delivery vehicle.

**Fig 2:**
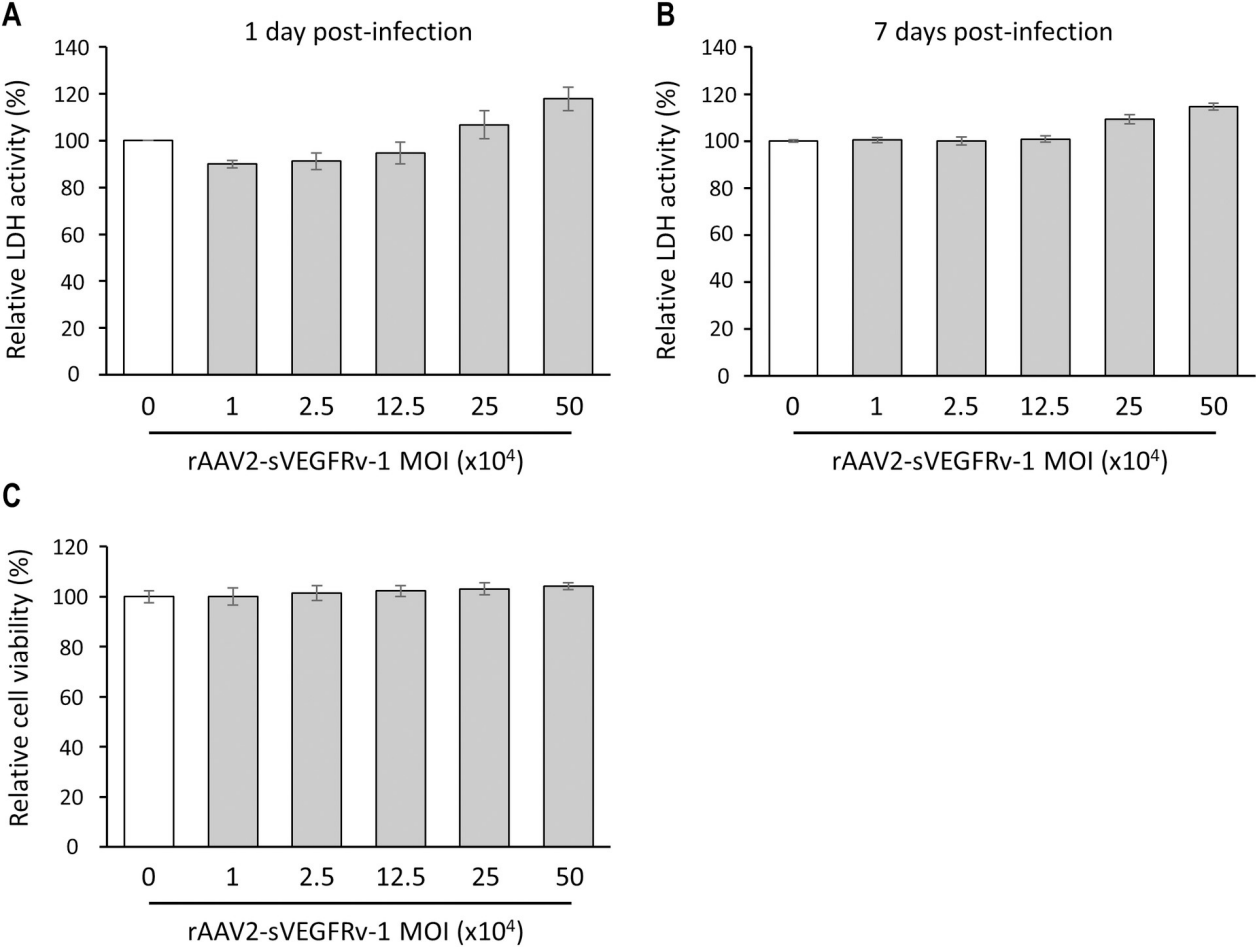
rAAV2-sVEGFRv-1 treatment does not result in cytotoxicity. Potential cytotoxic effects were evaluated via LHD activity assays on both shorter (a) and longer in vitro timescales (b), in addition to a cell viability assay (c), which helped demonstrate the safety of the therapeutic virus vector. Data represented as mean ± SEM (n = 3).

In order to then explore its safety profile, the effects of rAAV2-sVEGFRv-1 were examined via LDH cytotoxicity assays and a cell viability assay using increasing amounts of the virus vector. Relative to mock-treated control cells, cytotoxic effects were not observed until the number of viral genomes applied to the HUVECs was 10 times greater than the amount used throughout the rest of the study as the experimental infectious dose (Fig 2A), and the measured increases in LDH activity 1 day post-infection for cells treated at 250,000 MOI (106.8 ± 6.05) and 500,000 MOI (117.9 ± 5.00) were not markedly greater than that of the control group. Nearly identical results were obtained from HUVECs assayed 7 days after rAAV2-sVEGFRv-1 infection (Fig 2B), with cells treated at 250,000 MOI and 500,000 MOI exhibiting LDH activity levels of 109.3 ± 1.93 and 114.6 ± 1.52, respectively, compared to control cells. The therapeutic virus vector, meanwhile, did not have any significant effect on cell proliferation relative to the mock-treated control group (Fig 2C).

### rAAV2-sVEGFRv-1 interacts with VEGF-A, VEGF-B, and PlGF

**Fig 3:**
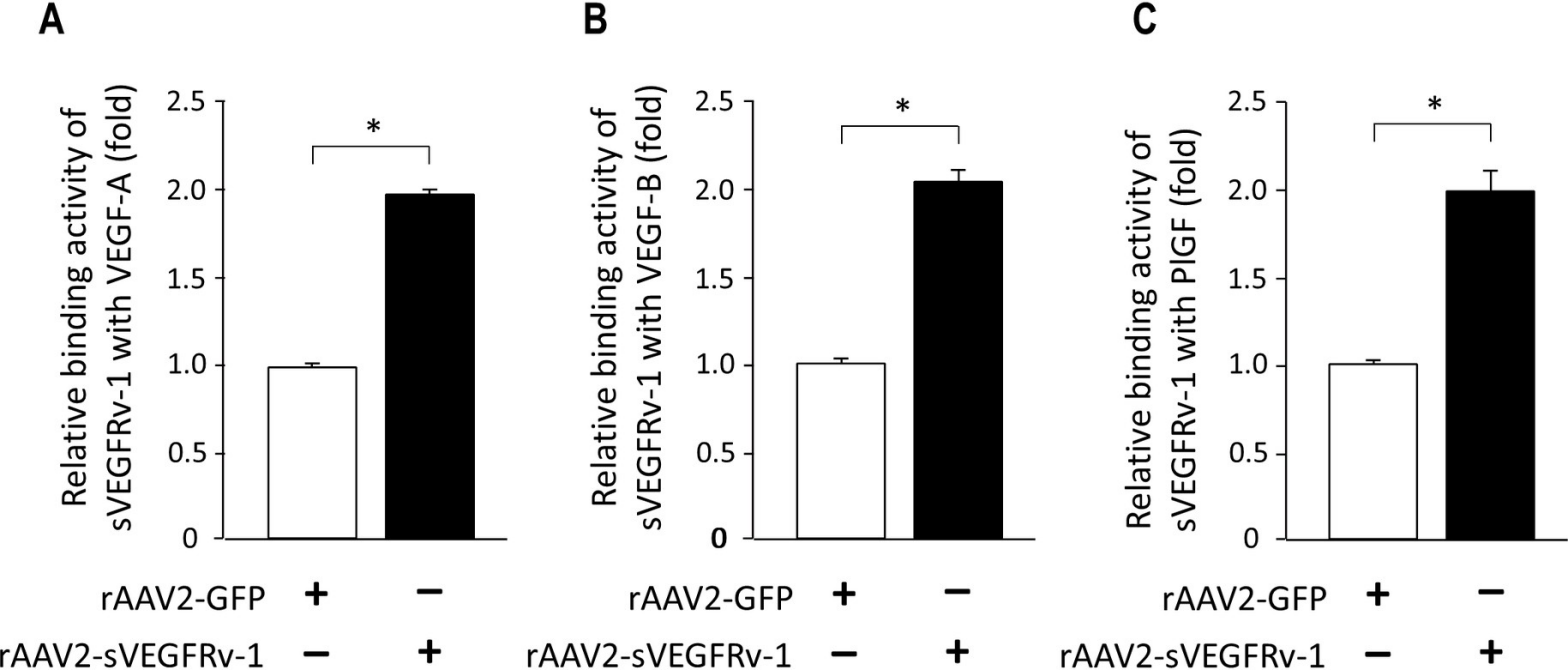
rAAV2-sVEGFRv-1 ligand binding. The ability of sVEGFRv-1 to interact with the natural binding partners of VEGFR1, including VEGF-A (a), VEGF-B (b), and PlGF (c) was determined via ELISA. Data represented as mean ± SEM (n = 3). * p < 0.05.

Having demonstrated that rAAV2-sVEGFRv-1 is able to effectively express the desired product encoded by its transgene, the activity of the resulting protein was then explored, particularly as it relates to its interactions with the natural binding partners of VEGFR1. HUVECs were infected with the therapeutic virus vector for 72 hours before ELISA binding assays were performed in the presence of one of the following members of the VEGF family: VEGF-B, VEGF-A, or PlGF, with the former only binding to VEGFR1, whereas the latter two bind both VEGFR1 and VEGF receptor-2 (VEGFR2). VEGF-A, additionally, is the VEGF family member most associated with pathological NV. Relative to the level of interaction observed in mock-treated control cells, it was demonstrated that the transgene product of rAAV2-sVEGFRv-1 binds with all three assayed ligands to a significant extent. Specifically, sVEGFRv-1 interacted with VEGF-B, its major binding partner, greater than twofold (2.085 ± 0.083, n = 3) the amount that occurred with the control group (Fig 3B). Similar results were observed with VEGF-A (1.888 ± 0.050, n = 3) (Fig 3A) and PlGF (1.803 ± 0.044, n = 3) (Fig 3C), demonstrating the potential therapeutic potency of the transgene product.

### Endothelial cell proliferation is reduced by rAAV2-sVEGFRv-1 treatment

**Fig 4:**
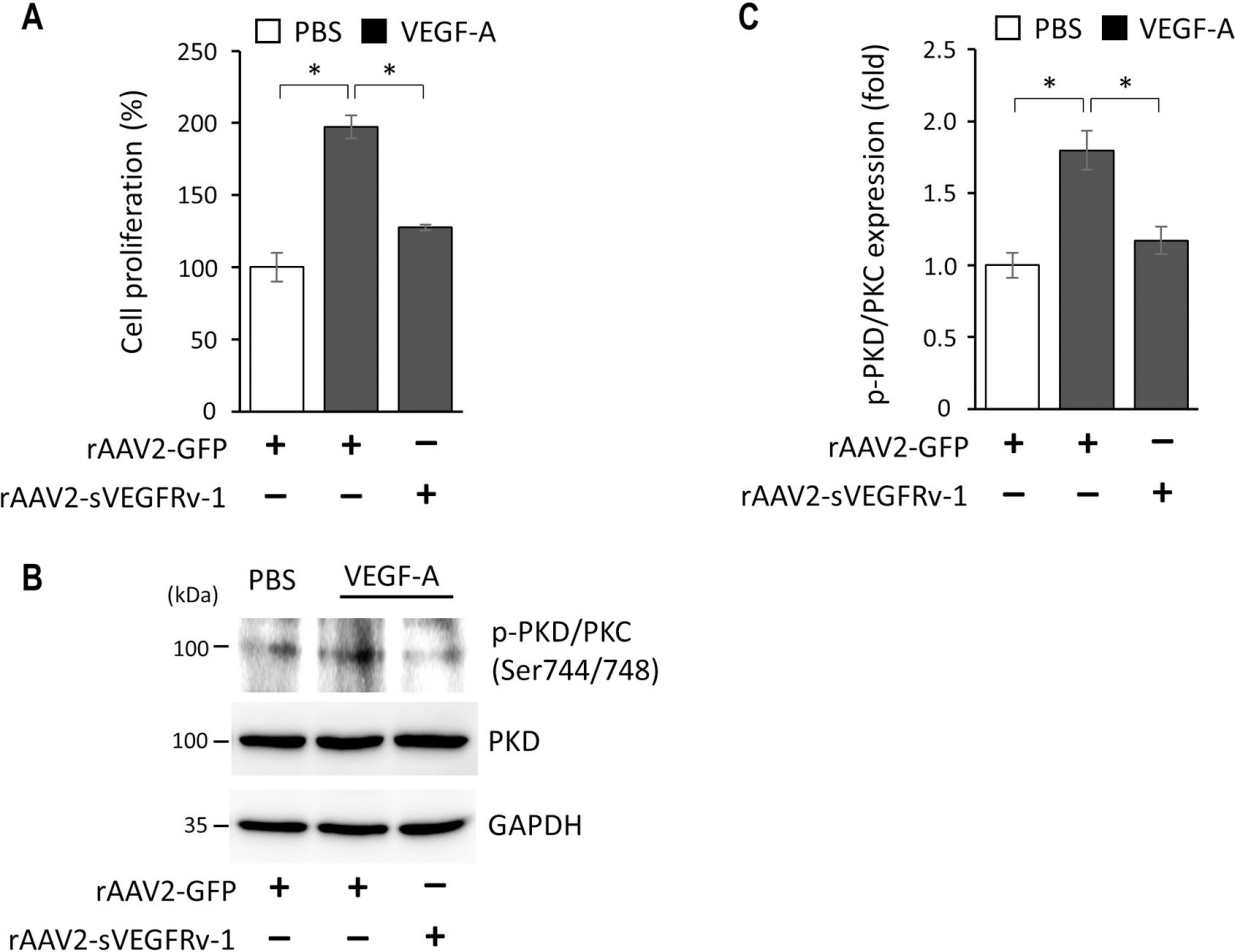
Effects of rAAV2-sVEGFRv-1 on HUVECs under VEGF-A stimulation. (a) The therapeutic virus vector reduced endothelial cell proliferation, a major contributor to the pathology of angiogenic ocular conditions induced by VEGF-A and via VEGFR2 activity, which may be due to its ability to block the phosphorylation (b) and activation of protein kinase D (PKD). (c) Quantification of the relative phosphorylation of PKD. Data represented as mean ± SEM (n = 3). * p < 0.05.

As such, it can be seen that the interaction between sVEGFRv-1 and VEGF-A mirrors the manner in which VEGFR1 acts as a trap to prevent VEGF-A from binding to VEGFR2 and activating its downstream signaling cascade. As the latter process is directly linked to angiogenic activity, the rAAV2-sVEGFRv-1 transgene product may thus abrogate some of the pathological processes associated with vascular ocular conditions, including endothelial cell proliferation. In order to examine the manner in which rAAV2-sVEGFRv-1 exerts its therapeutic effect with regard to VEGF-A-driven NV, an endothelial cell proliferation assay was performed using HUVECs. Determined as a percentage, a significantly increased amount of proliferation was found to occur in cells under VEGF-A stimulation treated with a control virus vector carrying an expression cassette containing a GFP reporter transgene (197.1 ± 8.00, n = 3) when compared to HUVECs infected with rAAV2-GFP and administered PBS (100.0 ± 9.82, n = 3). Meanwhile, rAAV2-sVEGFRv-1 treatment led to a marked reduction in HUVEC proliferation (127.5 ± 1.83, n = 3) in vitro, despite stimulation by VEGF-A (Fig 4A). Among the ways VEGF stimulates endothelial cell proliferation is through the phosphorylation and subsequent activation of protein kinase D (PKD). PKD, in turn, mediates ERK signaling and is also involved in VEGF-induced DNA synthesis via the downstream activity of VEGFR2 [25]. As shown (Fig 4B), PKD is present in HUVECs and VEGF-A stimulation leads to its phosphorylation. However, treatment with the therapeutic virus vector reduced PKD activation (Fig 4C) and may be a way by which rAAV2-sVEGFRv-1 effects its anti-angiogenic activity.

### rAAV2-sVEGFRv-1 maintains tight junction integrity by reducing VEGF-induced loss of ZO-1

**Fig 5:**
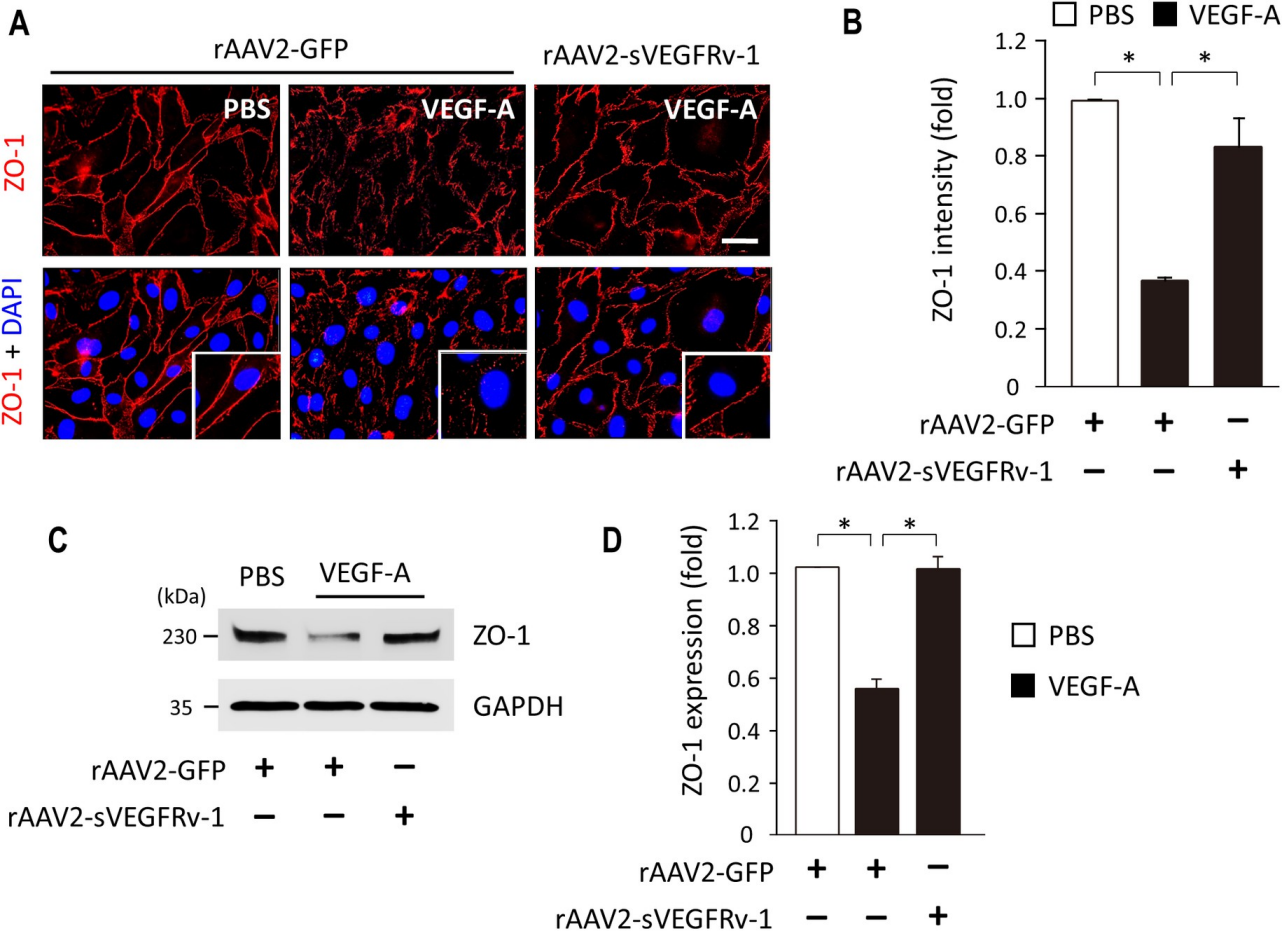
Tight junction protein 1 expression visualized in vitro. (a) Immunocytochemistry using HUVECs demonstrated that VEGF-A stimulation led to a relative absence of ZO-1 and affected its localization. These effects were mainly protected against by rAAV2-sVEGFRv-1 treatment, with the intensity of the fluorescence being quantified (b). Western blot (c) and the quantification thereof (d) was then used to confirm ZO-1 expression levels. Scale bar = 10 μm. Data represented as mean ± SEM (n = 3). * p < 0.05.

An additional process by which VEGF activity contributes to the pathophysiology of vascular conditions of the eye is by increasing cell permeability, which may lead to the development of leaky vessels and potential vision loss. ZO-1, which is also known as tight junction protein 1, is among the main structural proteins of tight junctions and a major component the blood-retina barrier (BRB) [26], whose disruption leads to the development of vascular leakage [27]. ZO-1 expression was impaired in HUVECs treated with rAAV2-GFP and stimulated with VEGF-A (Fig 5C), whereas cells infected with rAAV2-sVEGFRv-1 under VEGF-A stimulation had ZO-1 levels similar to that of unstimulated HUVECs treated with the control virus vector (Fig 5D). This was visualized via immunocytochemistry (Fig 5A), which showed, as a percentage relative to the control group (Fig 5B), that VEGF-A stimulation resulted in an observable reduction of ZO-1 in cells administered rAAV2-GFP (37.33 ± 0.006, n = 3), whereas rAAV2-sVEGFRv-1 treatment protected against the loss of ZO-1 (83.98 ± 0.096, n = 3), which may help maintain tight junction integrity and prevent vascular leakage.

### Anti-inflammatory activity of rAAV2-sVEGFRv-1

**Fig 6:**
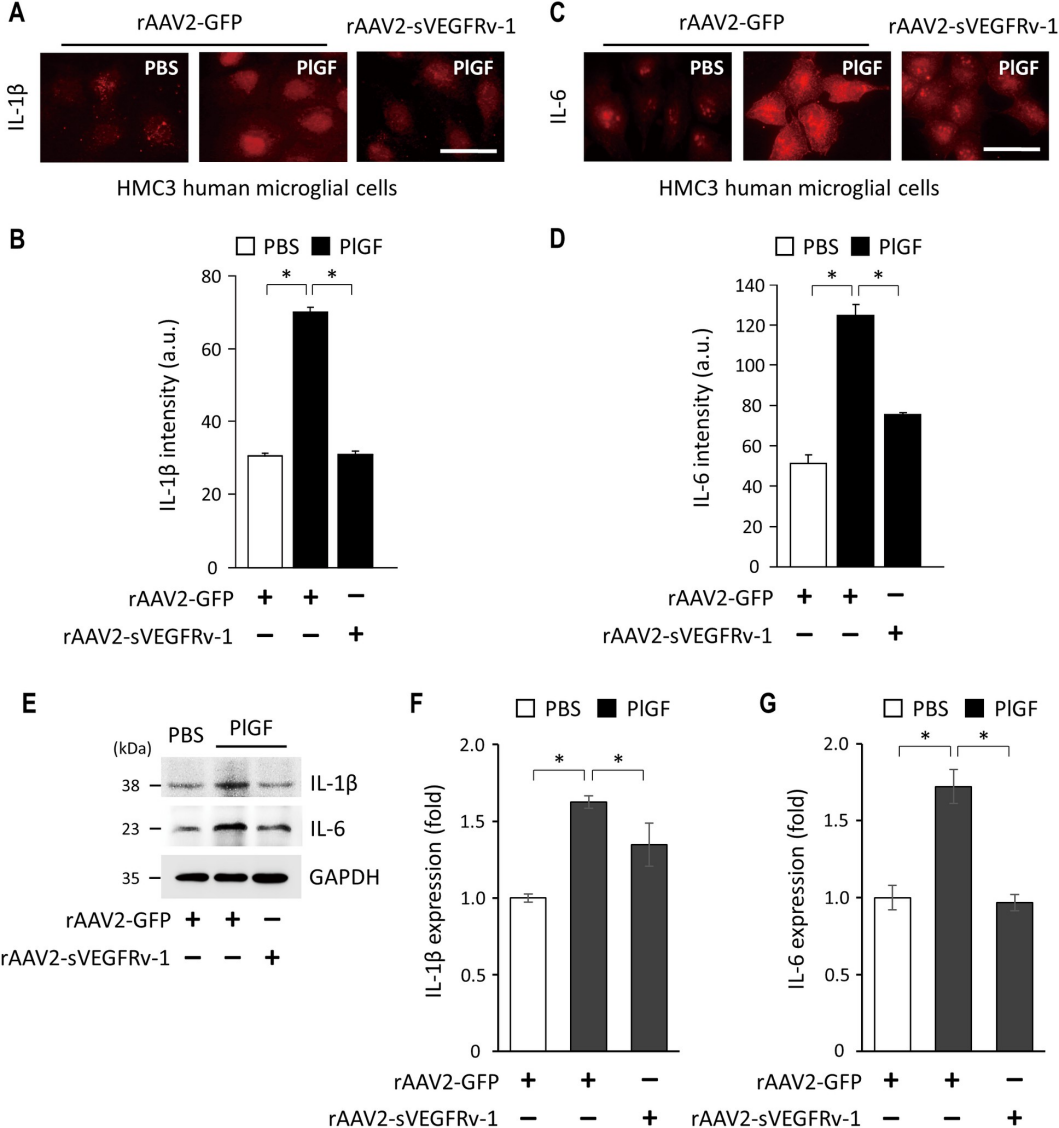
Effects of rAAV2-sVEGFRv-1 on interleukin production. HMC3 microglial cells were used to show that the therapeutic virus may be anti-inflammatory, as rAAV2-sVEGFRv-1 was able to protect against increases seen in IL-1β (a, b) and IL-6 production (c, d) induced by PlGF in cells treated with rAAV2-GFP, as compared to unstimulated control cells. (e) Western blot of the respective cytokine expression levels and the quantifications (f, g) thereof. Scale bars = 10 μm. Data represented as mean ± SEM (n = 3). * p < 0.05.

While VEGF drives the development of leaky vessels and subsequent NV leading to vision loss observed in the latter stages of AMD and DR, it also plays a role in mediating inflammation, which is observed early on in the pathophysiology of both conditions and has a major effect on their progression. Given the influence, particularly in the diseased eye [28], microglia have upon the production of immune-related factors, including pro-inflammatory cytokines IL-1β and IL-6 [5], human microglial HMC3 cells were used to elucidate the manner in which the therapeutic virus vector exerts its anti-inflammatory effects. PlGF, which itself is linked to IL-1β and IL-6 secretion [21], was used to stimulate HMC3 cells infected with either rAAV2-sVEGFRv-1 or a control virus vector. Relative to unstimulated control cells treated with rAAV2-GFP (30.85 ± 0.71, n = 3), PlGF administration led to a significant increase (70.42 ± 1.30, n = 3) in IL-1β secretion, as determined by measuring the intensity of the fluorescence of cells visualized using immunocytochemistry (Fig 6A). On the other hand, cells treated with both rAAV2-sVEGFRv-1 and PlGF (31.23 ± 1.03, n = 3) had IL-1β levels similar to that of the control group (Fig 6B). A similar pattern of results was obtained with regard to IL-6 (Fig 6C), wherein secretion of the pro-inflammatory cytokine was greatly increased (Fig 6D) in cells infected with the control virus vector and stimulated with PlGF (125.04 ± 5.30, n = 3), whereas rAAV2-sVEGFRv-1 treatment resulted in a markedly less pronounced increase in IL-6 levels (75.84 ± 0.93, n = 3) compared to the control group (51.57 ± 3.98, n = 3). These effects upon the expression of the cytokines were confirmed via Western blot (Fig 6E), with the results quantified (S1 Table) for both IL-1β (Fig 6F) and IL-6 (Fig 6G).

## Discussion

Here, we explored the in vitro activity of rAAV2-sVEGFRv-1 to elucidate the mechanisms by which it exerts its therapeutic efficacy in vivo, as exhibited in mouse models of nvAMD [22] and DR [23]. By showing in HUVECs that its transgene product interacts with the natural ligands of VEGFR1, we were able to demonstrate the therapeutic potency of rAAV2-sVEGFRv-1 through its ability to bind VEGF-A, VEGF-B, and PlGF (Fig 3). As a result of these interactions, the therapeutic virus vector was able to protect against the proliferative effects of VEGF-A (Fig 4), as well as the latter’s ability to induce tight junction dysfunctions, visualized via immunocytochemistry by a reduction in the loss of ZO-1 (Fig 5). These processes are associated with angiogenesis and vascular permeability in the pathophysiology of nvAMD and DR, for which VEGF-A acts as the main driver through its interaction with VEGFR2 [5, 6]. However, by additionally showing in HMC3 microglial cells that rAAV2-sVEGFRv-1 administration reduced the secretion of the pro-inflammatory cytokines IL-1β, a master regulator of inflammation [26], and IL-6, which is also highly influential with regard to inflammatory processes [29] (Fig 6), we are able to lend further support to the growing evidence regarding the importance of VEGFR1 signaling to pathological processes associated with angiogenic ocular conditions [5].

Although the complexities of the overlapping and interconnected interactions between VEGF family members and their receptors, as well as the context-dependence thereof [5], tend to complicate matters, the decoy receptor function of VEGFR1 with respect to VEGF-A [5, 20] is the most straightforward manner in which rAAV2-sVEGFRv-1 is able to effect its anti-angiogenic activity. Utilizing its greater affinity for VEGF-A than VEGFR2 [20], VEGFR1 is able to bind the former to sequester it from interacting with the latter and activating the VEGFR2 pathway [5, 30], the canonical process by which pathological angiogenesis occurs in AMD and DR [5]. By doing so, VEGFR1 serves as a trap for VEGF-A [18], a characteristic it shares with its soluble variant [19, 24, 31]. This leads to a reduction in endothelial cell proliferation, a process in which protein kinase D plays a role upon being activated by VEGF-A via phosphorylation [25], which we were able to explore here in HUVECs (Fig 4). These results comport with our previous work in a laser-induced mouse model of CNV, in which rAAV2-sVEGFRv-1 was shown to reduce the extent to which CNV lesions occurred [22]. The anti-angiogenic activity of sFlt-1 is also observed in the cornea, whose avascularity is maintained by the actions of the soluble receptor despite the presence of VEGF-A, both of which are highly expressed in corneal tissues [5, 32, 33].

Given that ligand access and availability play major roles in VEGF receptor signaling activation, it may seem that the ability of VEGFR1 to bind VEGF-B and PlGF would be deleterious, resulting in greater amounts VEGF-A being left free to interact with VEGFR2 [5]. However, VEGFR1 signaling may itself potentially play important roles in pathological angiogenesis [33, 34]. VEGF-B is associated with nonvascular processes associated with DR and nvAMD, including the induction of inflammatory responses and inhibiting apoptosis [34]. Meanwhile, there is growing evidence of the importance of PlGF, which is structurally the most similar to VEGF-A among VEGF family members [5], to the pathophysiology of DR and nvAMD [21].

Studies have shown that PlGF is involved in pathological angiogenesis [33, 35], and though the precise mechanisms through which it influences the angiogenic process have yet to be elucidated [6], the connection has long been observed [36]. An important consideration here may be the ability of PlGF to attract inflammatory cells, including monocytes [35], macrophages [21], and leukocytes [36], and the link between inflammatory cell infiltration and angiogenesis [21, 35]. PlGF may be upregulated in angiogenic ocular conditions and therapeutically beneficial effects have been observed upon its modulation [21, 36], such as reductions in the sizes of laser-induced CNV lesions and protection against vascular leakage in diabetic mice [21]. The latter may be due to the effect that PlGF has upon tight junctions in the retina [37], and a PlGF-targeting therapeutic has since been developed for diabetic macular edema (DME) [35], a complication of DR linked with vascular permeability that is also the main cause of DR-associated blindness [12].

VEGF-A also induces the development of leaky vessels and was alternatively called vascular-permeability factor (VPF) during the initial stages of its research and discovery [18]. Increased vascular permeability is an important early characteristic of pathological angiogenesis [38], with intercellular integrity maintained by junctional proteins like ZO-1 [39], a key tight junction complex scaffold protein that can be rapidly phosphorylated by VEGF-A [40]. ZO-1 loss has been linked to dysfunctions of the retinal pigment epithelium (RPE), which may in turn lead to VEGF-A overexpression and AMD development [41]. Meanwhile, aberrant ZO-1 expression is connected to RPE cell loss, breakdowns in the BRB, and retinal degeneration leading to DR [42]. We were able to previously demonstrate in a STZ-induced mouse model that rAAV2-sVEGFRv-1 reduces vascular leakage, retinal pericyte loss, and the development of acellular capillaries [23]. This may be due to the therapeutic virus vector’s ability to preserve ZO-1 expression and functionality (Fig 5), particularly given that PlGF has also been shown to disrupt ZO-1 and tight junction integrity [37].

As NV driven by VEGF-A is responsible for the blindness that occurs with AMD and DR [3, 5], clinical trials of the most widely-used anti-VEGF therapeutics, as well as comparative studies among them, have regarded visual gains as an accepted metric of therapeutic efficacy [5, 35]. However, being multifactorial disorders [43], increasing attention is being paid to the overarching influence of pathological processes like inflammation [5, 9], including the effects the interplay between pathophysiological conditions, VEGFR1 ligands, pro-inflammatory factors, and the cells from which they originate, have upon the pathogenesis and progression of AMD and DR [7, 9, 44, 45]. In fact, the effects of inflammation are such that research is currently being pursued on a number of anti-inflammatory therapeutics with novel targets acting via non-VEGF pathways [43]. The significant role played by the VEGF family in inflammation, particularly the recruitment of monocytes and leukocytes by VEGFR1 ligands [35], means that rAAV2-sVEGFRv-1 being able to protect against inflammatory cell infiltration by macrophages and leukocytes [22] addresses this influential aspect of angiogenic ocular disorders like AMD and DR.

Leukocytes, including macrophages and microglia, are present in diseased retinas and help drive the pathogenesis [7, 9, 44] and early-stage progression of AMD through inflammasome and complement system activation. They have additionally been set forth as a potential therapeutic target for dry AMD [44]. Inflammasome expression was found to be upregulated in patient samples of both AMD subtypes [46], and upon activation, secrete IL-1β [44], which is involved in additional leukocyte recruitment [26, 28] and cytokine modulation. This includes the activation [44] and upregulation [26, 28, 47] of the pro-inflammatory IL-6, which has also been implicated in AMD pathogenesis [9, 44] and is produced by various leukocytes subpopulations in response to oxidative stress [26], a potential causative factor of AMD [27, 29]. Additional roles for IL-1β in pathological processes include increasing BRB permeability, affecting endothelial cell morphology and function, and the induction of angiogenesis, partially via IL-1β and VEGF-A upregulating one another in a self-amplifying feedback loop [26]. A similar association with VEGF-A also exists for IL-6 [29], with VEGF-A and IL-6 found to be correlated in AMD with CNV activity and CNV size, respectively [48]. IL-6 has also been linked to endothelium dysfunction [29], vascular leakage development [45], and the infiltration of leukocytes [26], monocytes, and T lymphocytes [45].

Elevated levels of IL-1β have also been observed in the serum [49], vitreous, and aqueous humor of DR [44] and DME [26] patients, with PDR patients showing an increase in PlGF [49] and IL-6 as well [29]. In DR, the activity of these pro-inflammatory cytokines has been associated with the induction of capillary blockage [49] and tight junction alternations [29], resulting in the development of DME, vascular leakage, and acellular capillary formation [49], the latter two of which are directly addressed by rAAV2-sVEGFRv-1 [23]. Meanwhile, PlGF inhibition has been shown to reduce the expression of cytokines, including IL-1β and IL-6, as well as other inflammatory markers, in addition to protecting against macrophage and microglia infiltration and tissue destruction [21]. Our in vitro results here (Fig 6), using a cell line from which the production of both pro- and anti-inflammatory cytokines is possible [50], may thus explain our in vivo observations of the anti-inflammatory effects of rAAV2sVEGFRv-1 [22].

By demonstrating that its transgene product is able to bind VEGF-A, VEGF-B, and PlGF, and exploring the resulting effects thereof, we were able to begin examining the manner of the therapeutic efficacy of rAAV2-sVEGFRv-1. Having shown that the therapeutic virus vector is anti-proliferative, protects against the loss of structural tight junction proteins, and reduces IL-1β and IL-6 levels, links may be made to the therapeutic activity it has previously exhibited in animal models of nvAMD and DR. Future studies to further develop rAAV2-sVEGFRv-1 as a human gene therapeutic include a long-term safety evaluation, which is currently underway, and an efficacy study in non-human primates.

## Supporting information

S1 Table Effect of PlGF stimulation on cytokine expression levels in HMC3 cells, normalized using GAPDH. (XLSX)

S1 Fig Microglia visualized in HMC3 cell culture via immunocytochemistry using Iba-1 antibody. (TIF)

S2 Fig Recombinant AAV constructs at an MOI of 25,000 are able to effectively infect HMC3 human microglial cells and successfully express their transgenes therein. (TIF)

S1 Raw images (PDF)

S2 Raw images (PPTX)
