# Supplementary figures and images for: An examination of the mechanisms driving the therapeutic effects of an AAV expressing a soluble variant of VEGF receptor-1
Source: PLoS One. 2024 Jul 11;19(7):e0305466. doi: 10.1371/journal.pone.0305466 (PMC11239064; doi:10.1371/journal.pone.0305466)

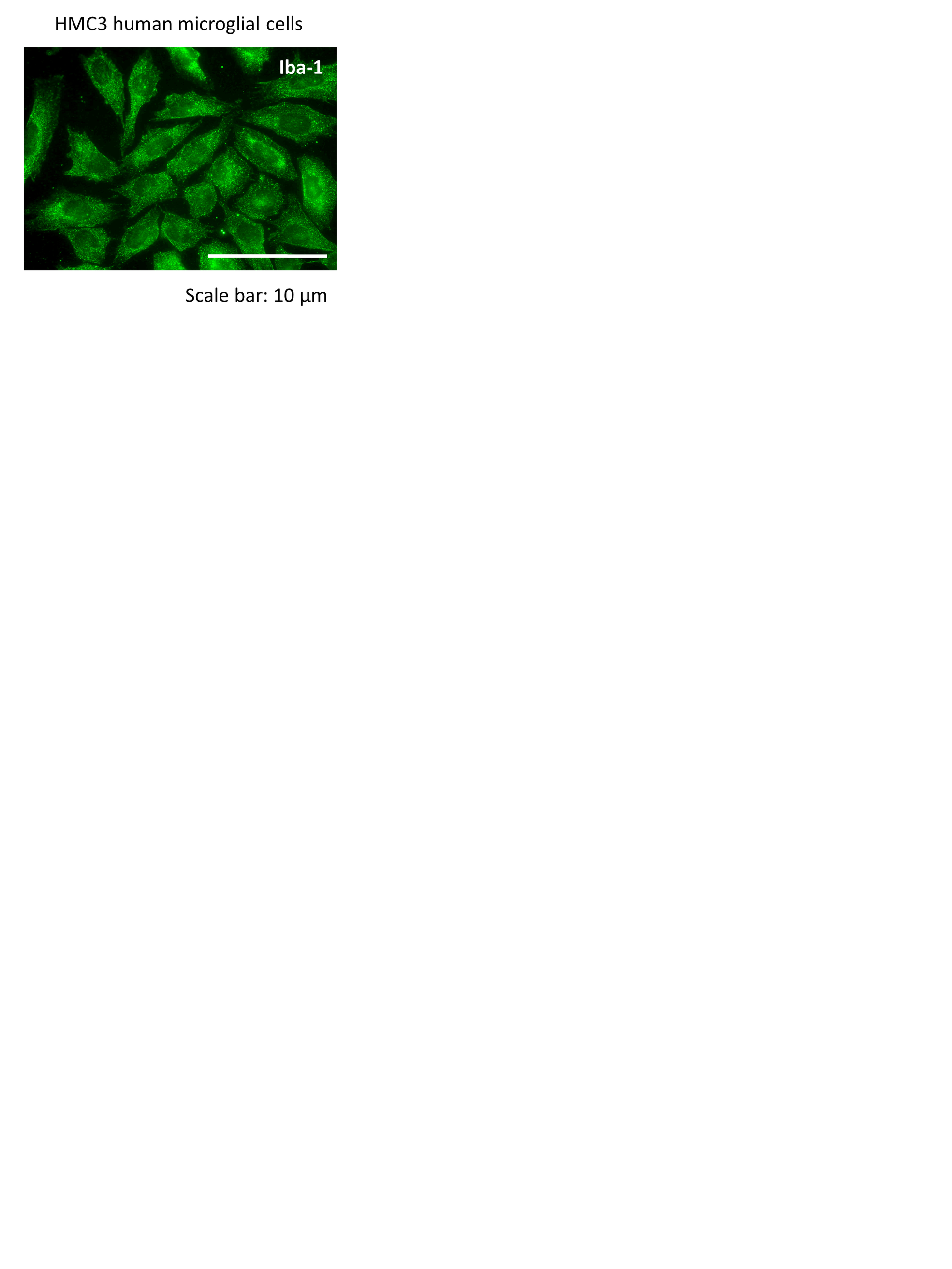

Supplement: S1 Fig — (TIF) [file pone.0305466.s002.tif]

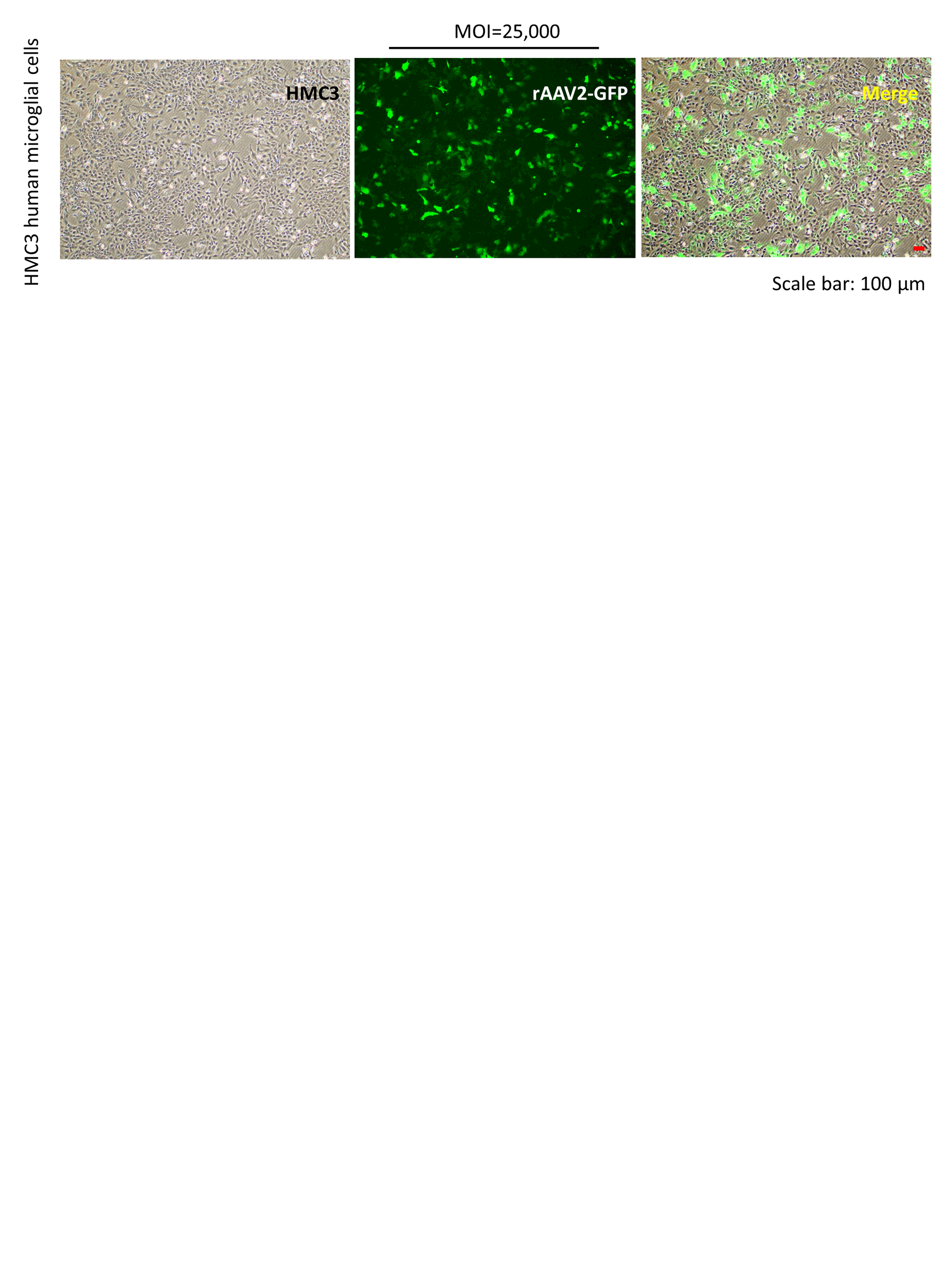

Supplement: S2 Fig — (TIF) [file pone.0305466.s003.tif]
