# Supplementary material for: An examination of the mechanisms driving the therapeutic effects of an AAV expressing a soluble variant of VEGF receptor-1
Source: PLoS One. 2024 Jul 11;19(7):e0305466. doi: 10.1371/journal.pone.0305466 (PMC11239064; doi:10.1371/journal.pone.0305466)

Figure 1B – RT-PCR – sVEGFRv-1 & β-actin

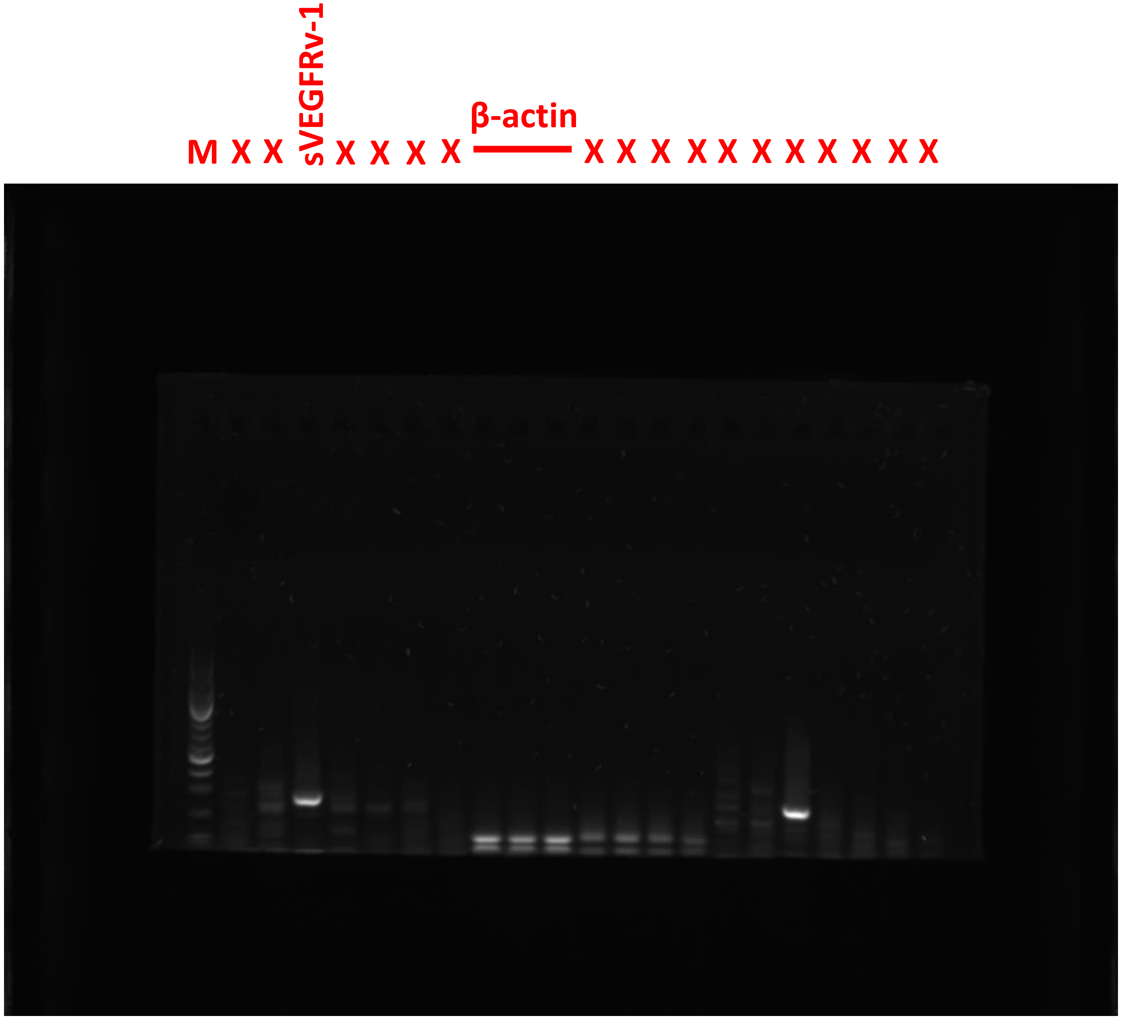

Figure 4B – Western blot – p-PKD/PKC

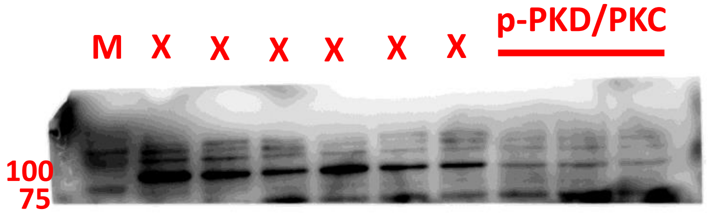

Figure 4B – Western blot – PKD

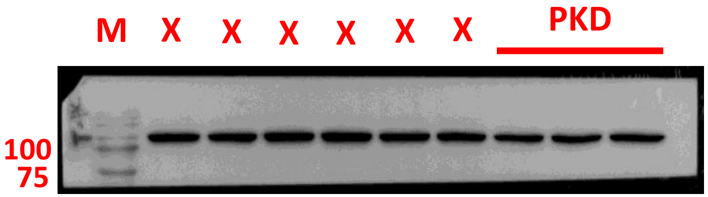

Figure 4B – Western blot – GAPDH

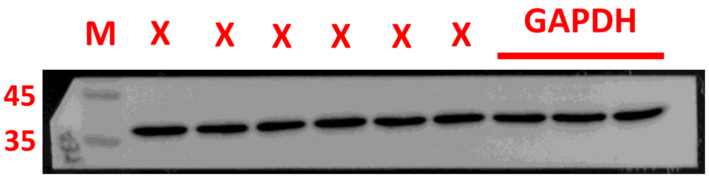

Figure 5C – Western blot – ZO-1

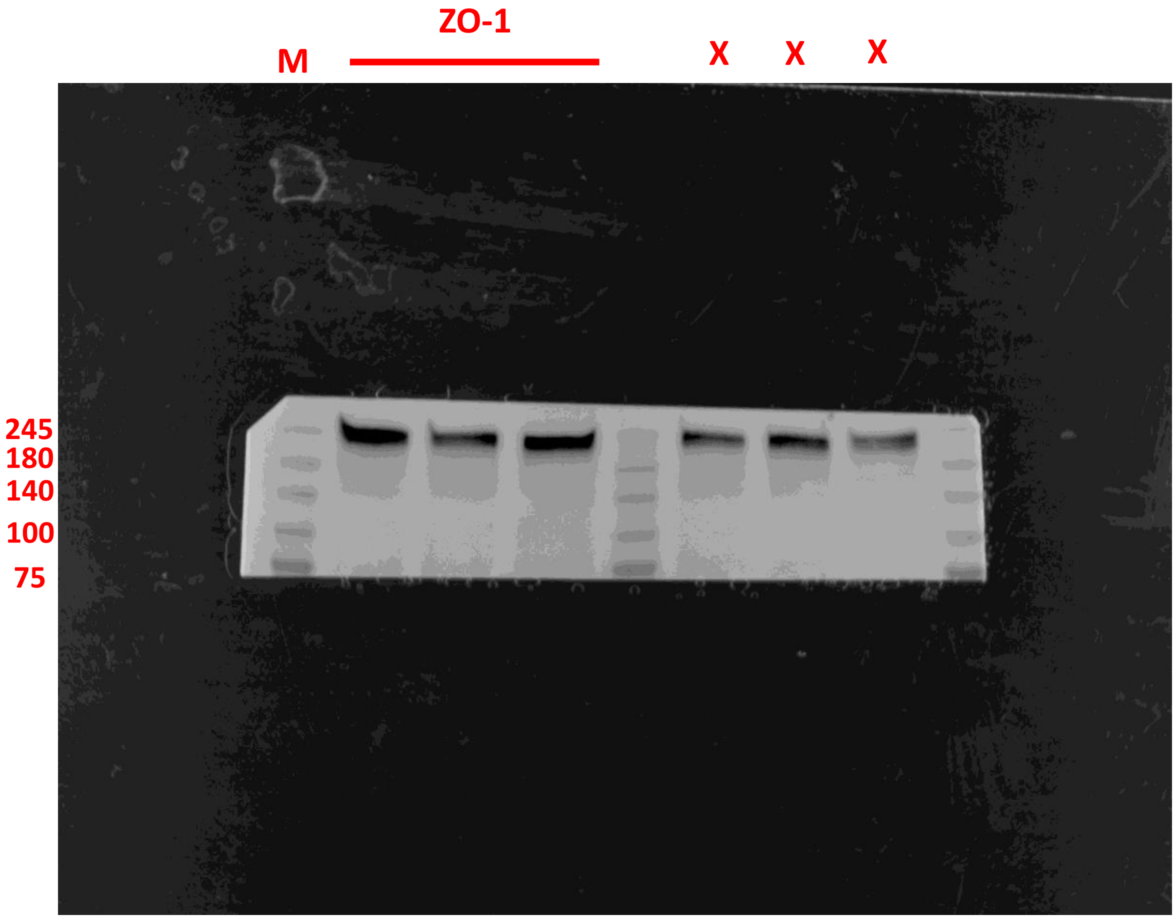

Figure 5C – Western blot – GAPDH

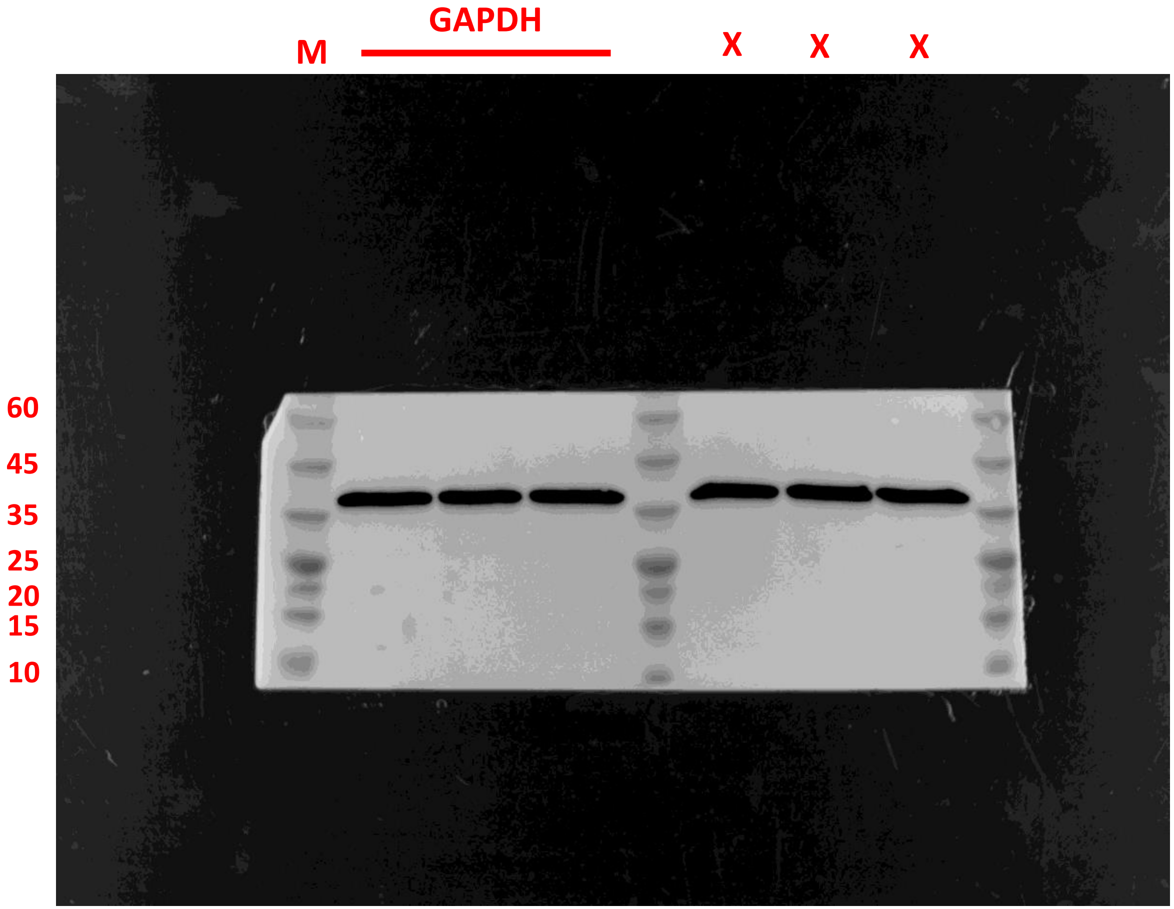

Figure 6E – Western blot – IL-1β

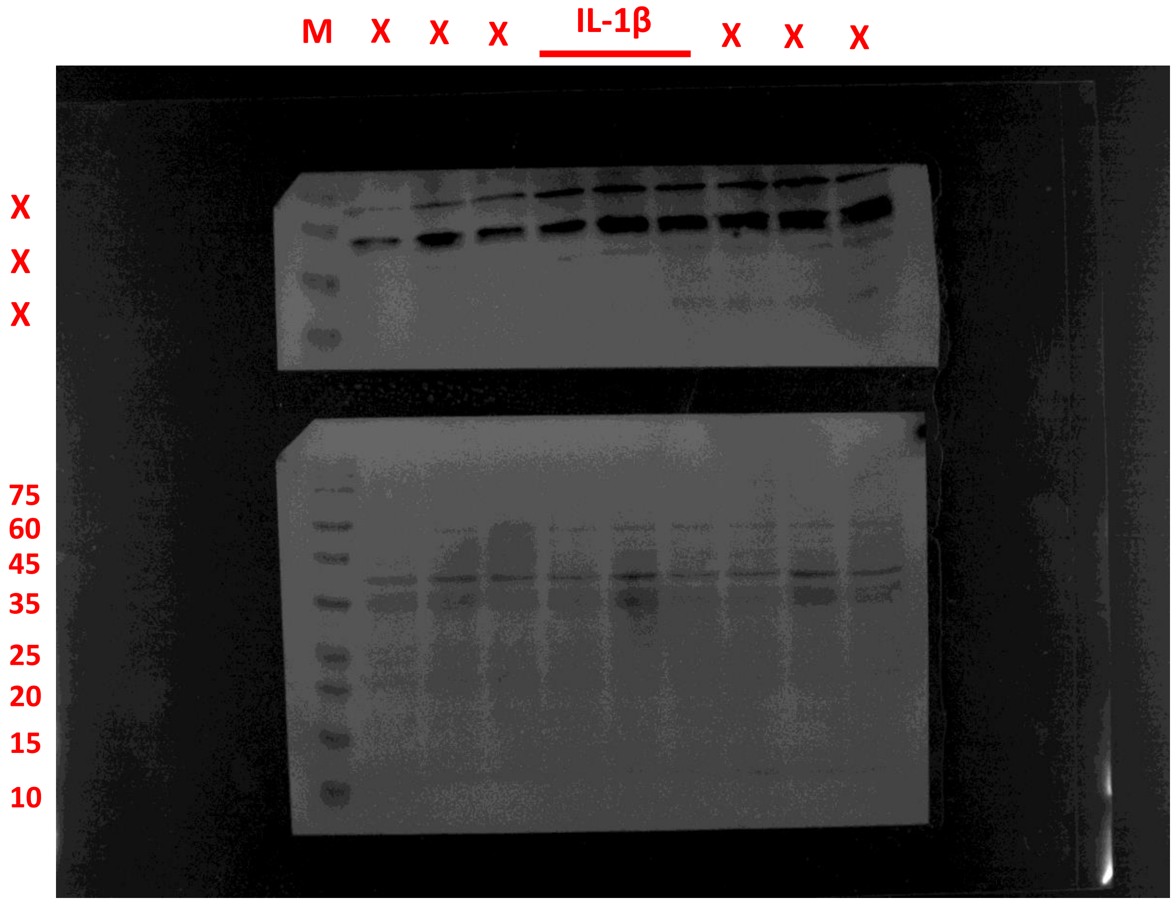

Figure 6E – Western blot – IL-6

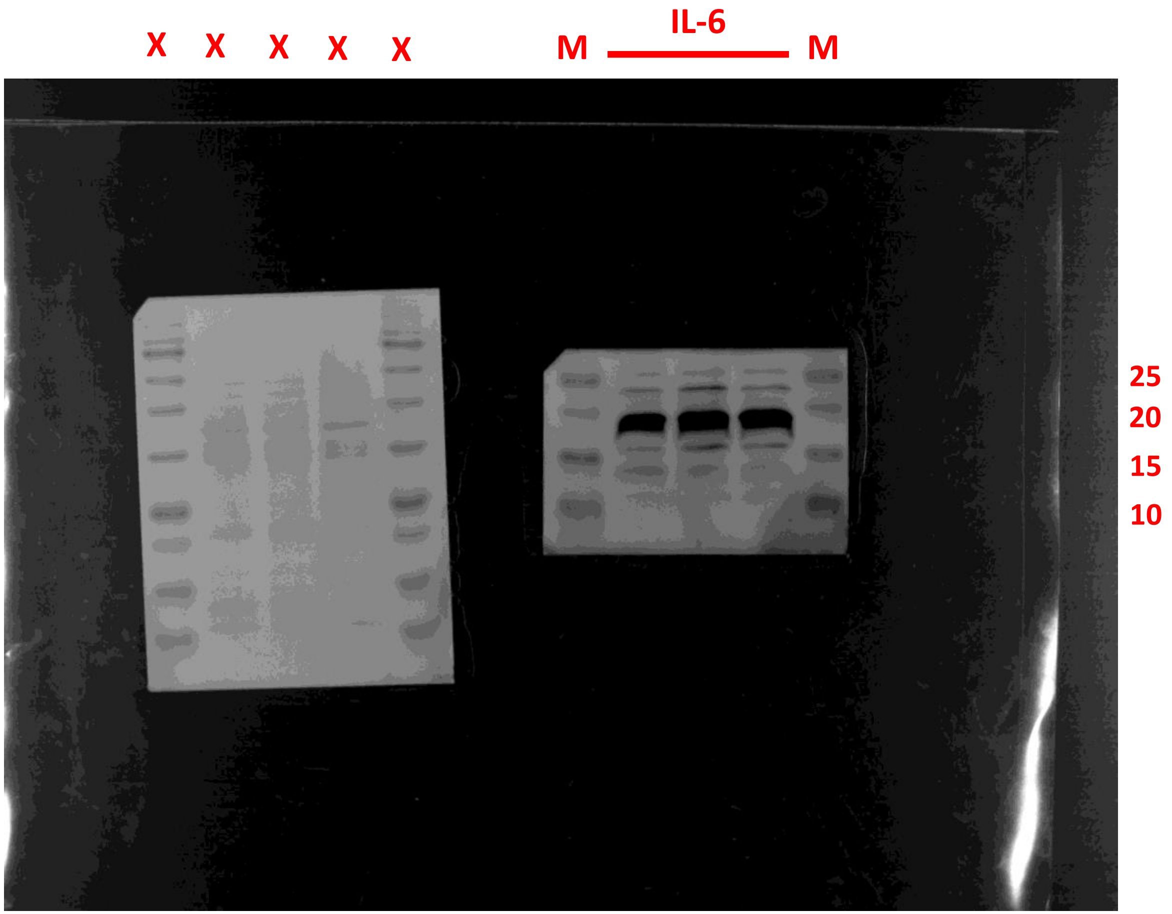

Figure 6E – Western blot – GAPDH

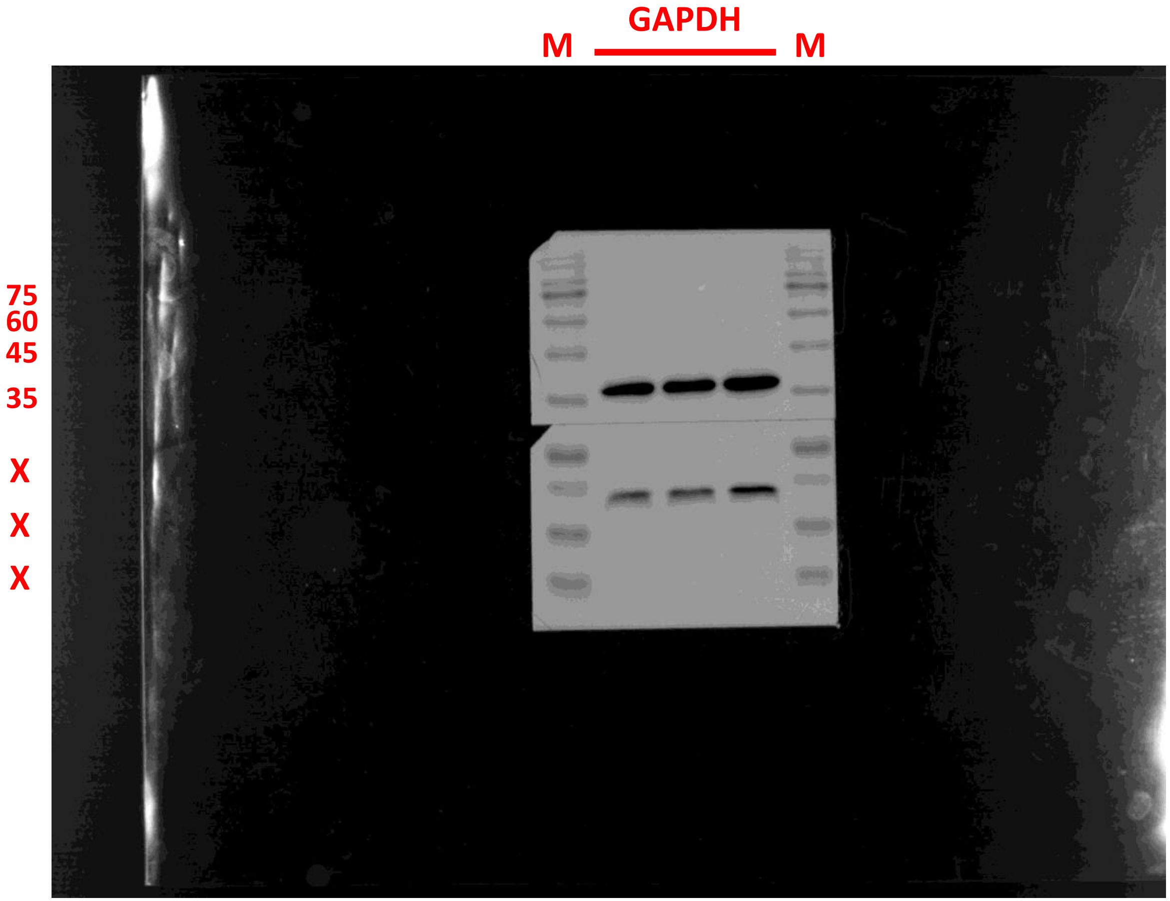

Supplement: S1 Raw images — (PDF) [file pone.0305466.s004.pdf]
