# Supplementary material for: An examination of the mechanisms driving the therapeutic effects of an AAV expressing a soluble variant of VEGF receptor-1
Source: PLoS One. 2024 Jul 11;19(7):e0305466. doi: 10.1371/journal.pone.0305466 (PMC11239064; doi:10.1371/journal.pone.0305466)
Supplement: S2 Raw images — (PPTX) [file pone.0305466.s005.pptx]

## Slide 1
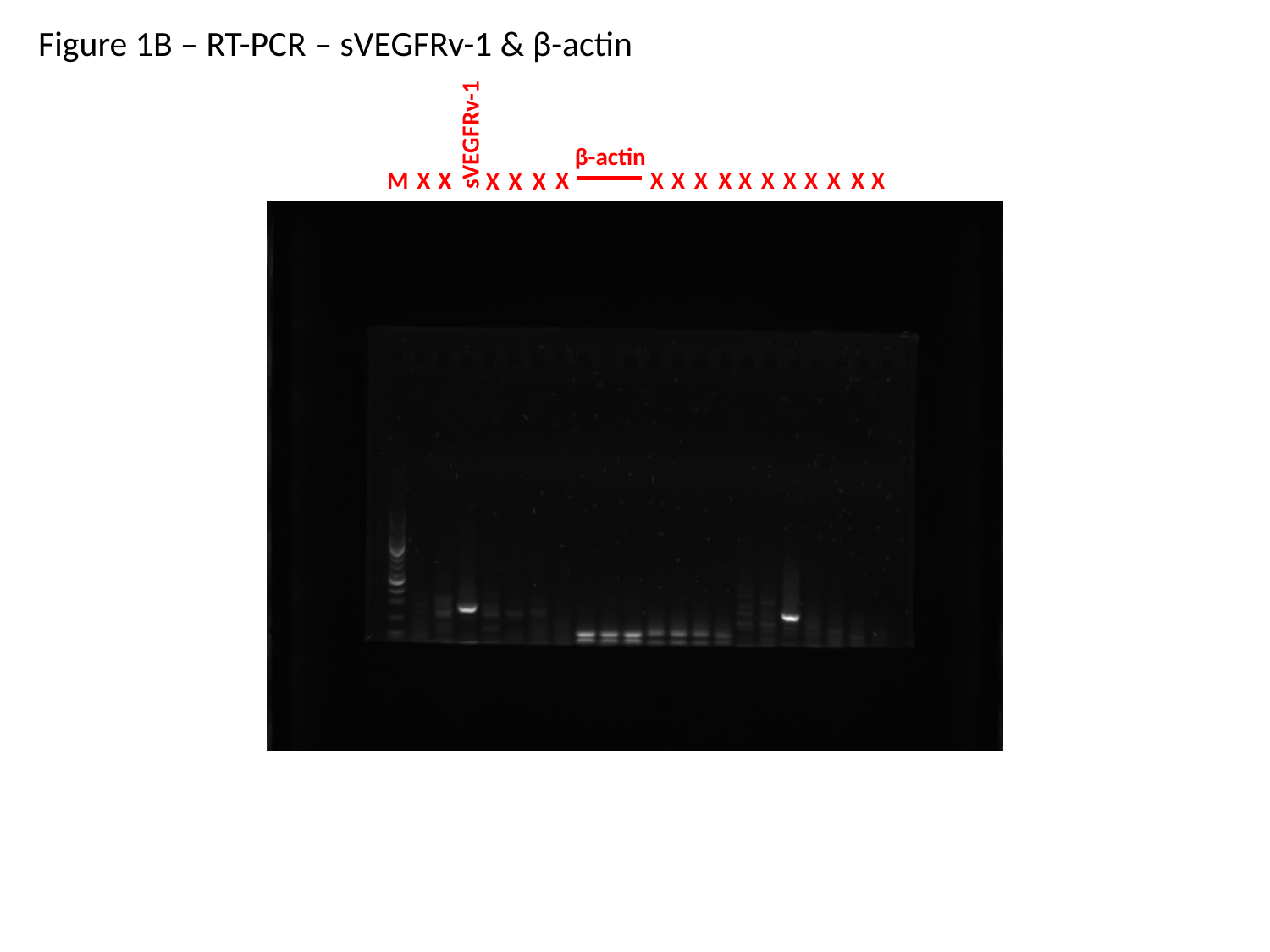

Figure 1B – RT-PCR – sVEGFRv-1 & β-actin
sVEGFRv-1
β-actin
X
X
X
X
X
X
X
X
X
X
X
X
X
X
M
X
X
X

## Slide 2
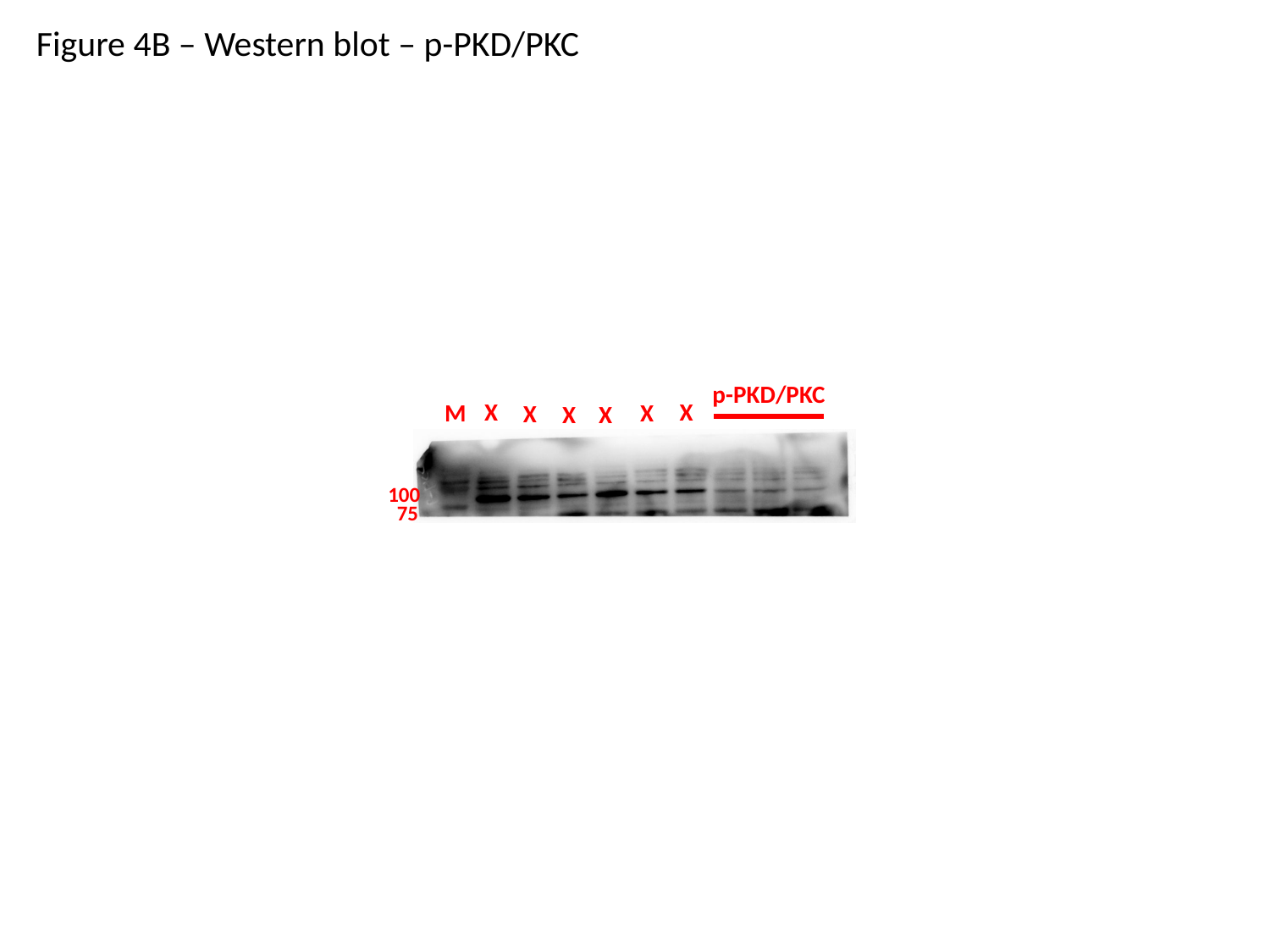

Figure 4B – Western blot – p-PKD/PKC
p-PKD/PKC
X
X
M
X
X
X
X
100
75

## Slide 3
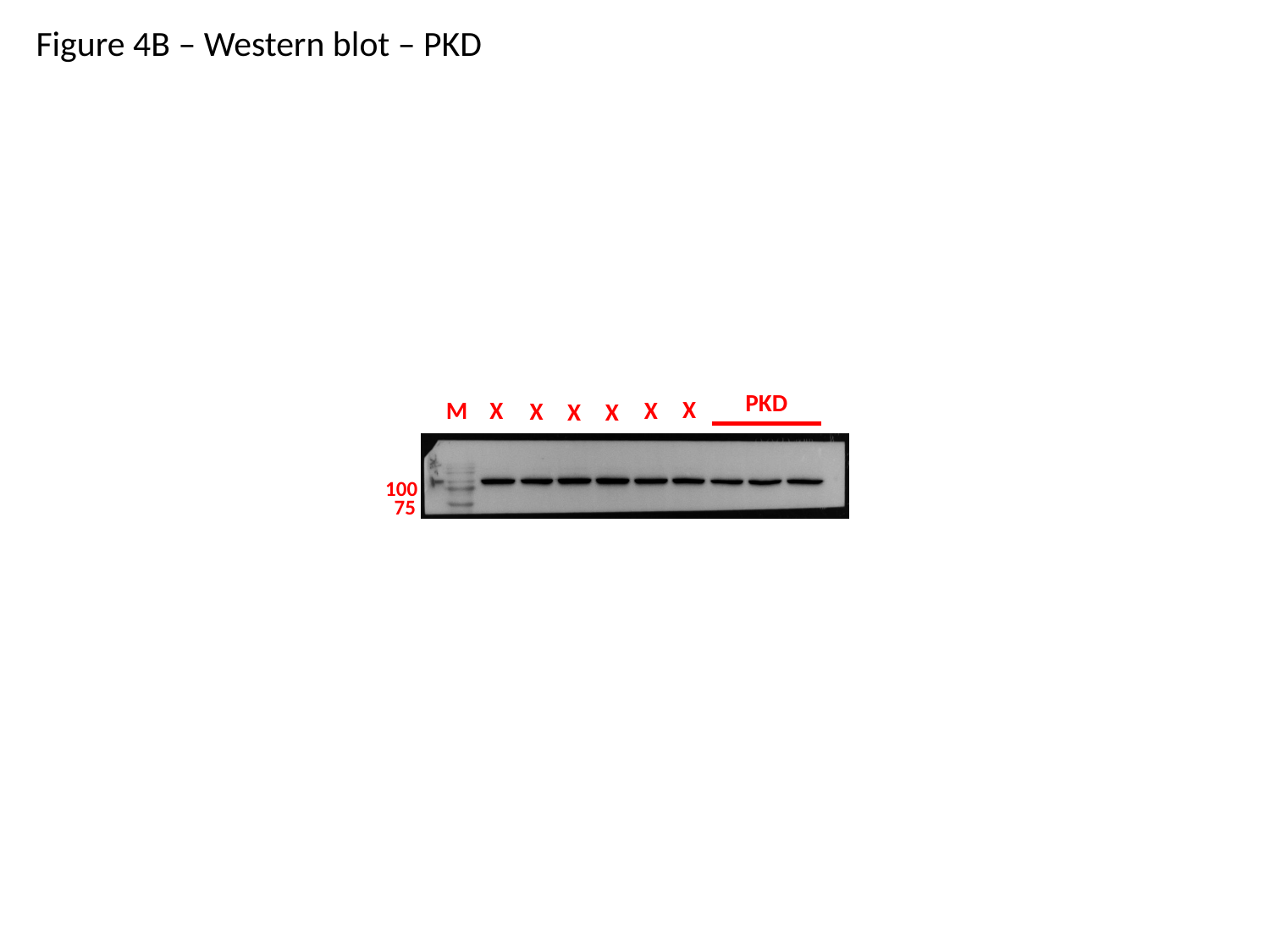

Figure 4B – Western blot – PKD
PKD
X
X
M
X
X
X
X
100
75

## Slide 4
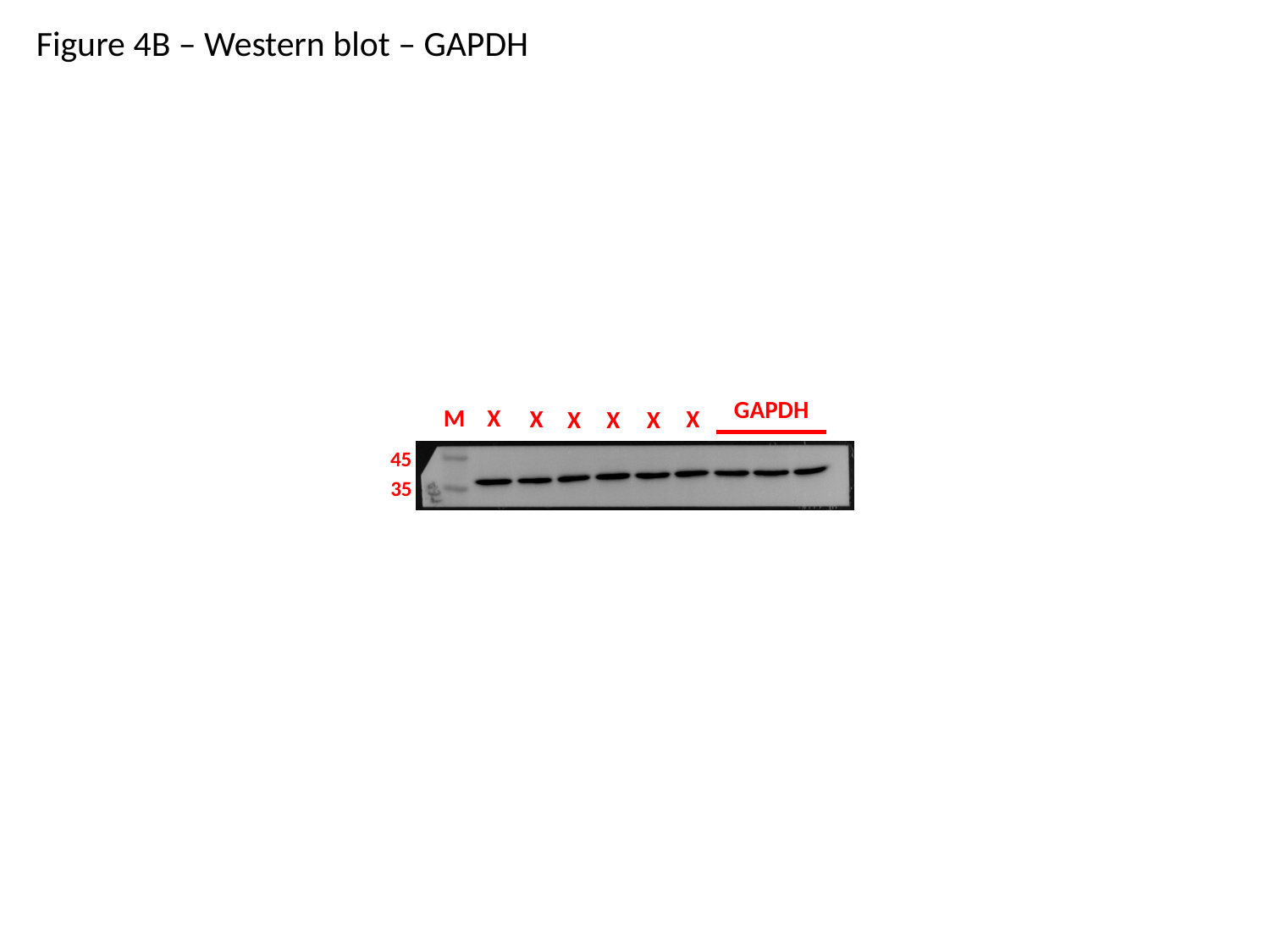

Figure 4B – Western blot – GAPDH
GAPDH
X
M
X
X
X
X
X
45
35

## Slide 5
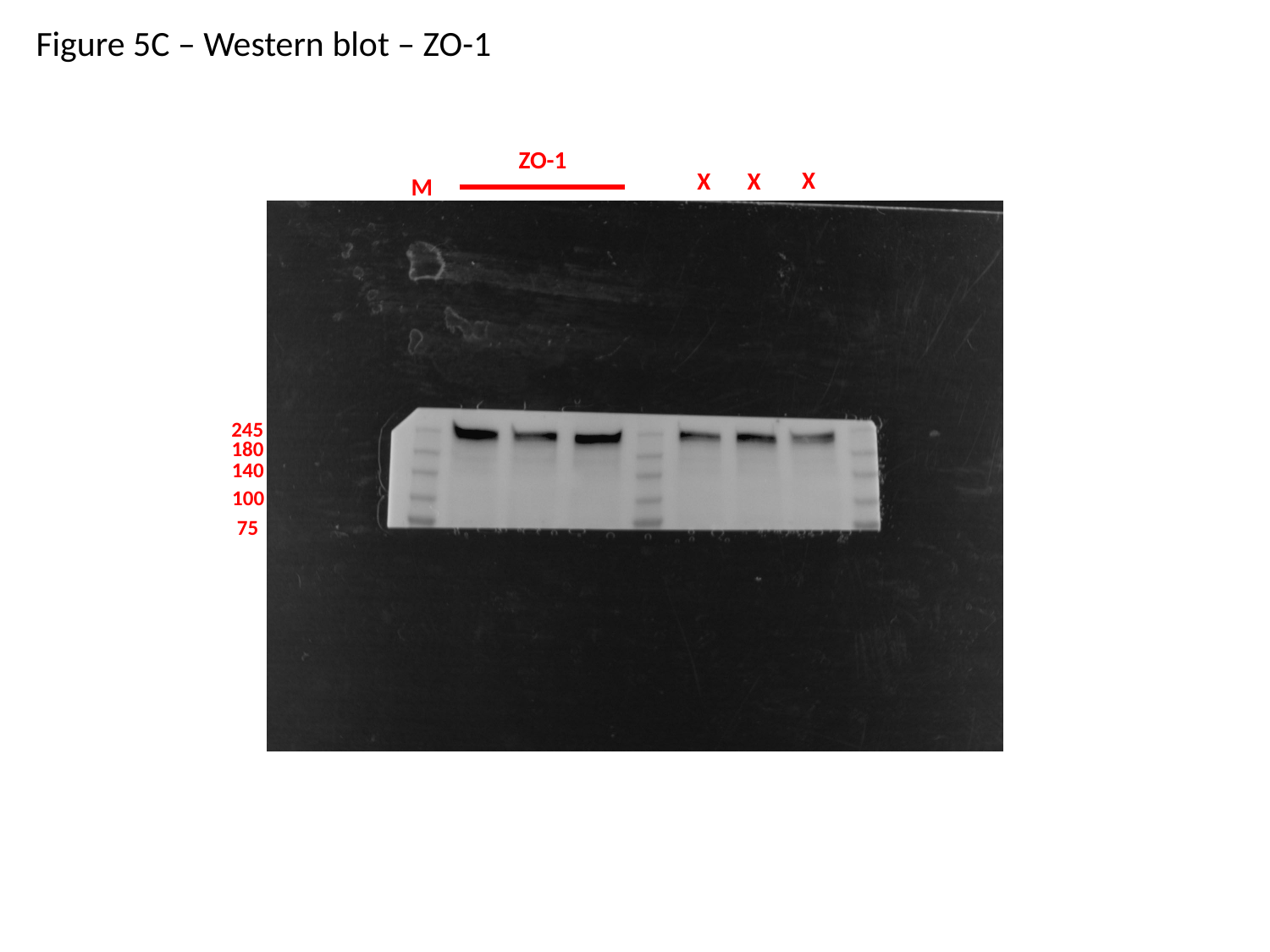

Figure 5C – Western blot – ZO-1
ZO-1
X
X
X
M
245
180
140
100
75

## Slide 6
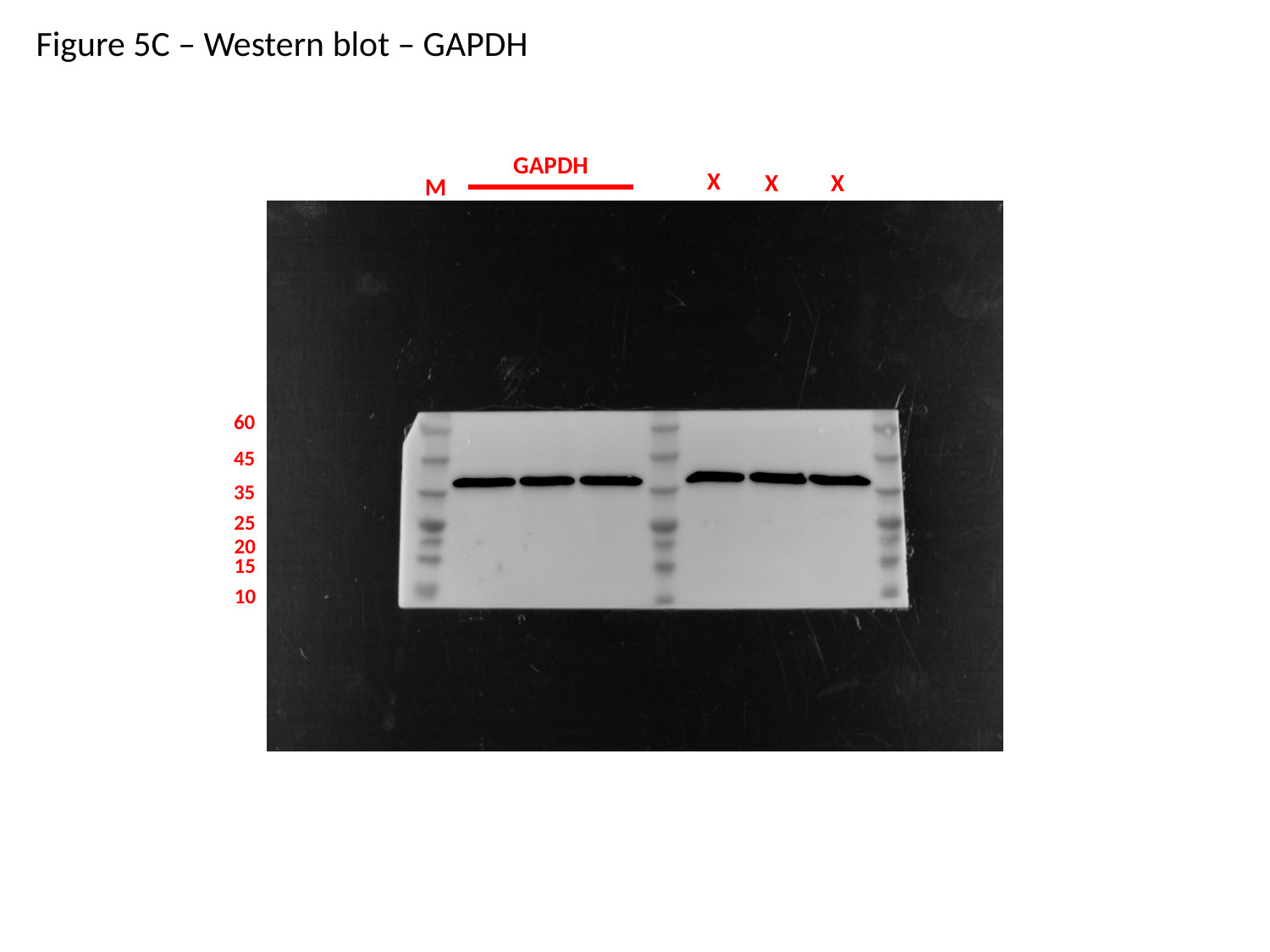

Figure 5C – Western blot – GAPDH
GAPDH
X
X
X
M
60
45
35
25
20
15
10

## Slide 7
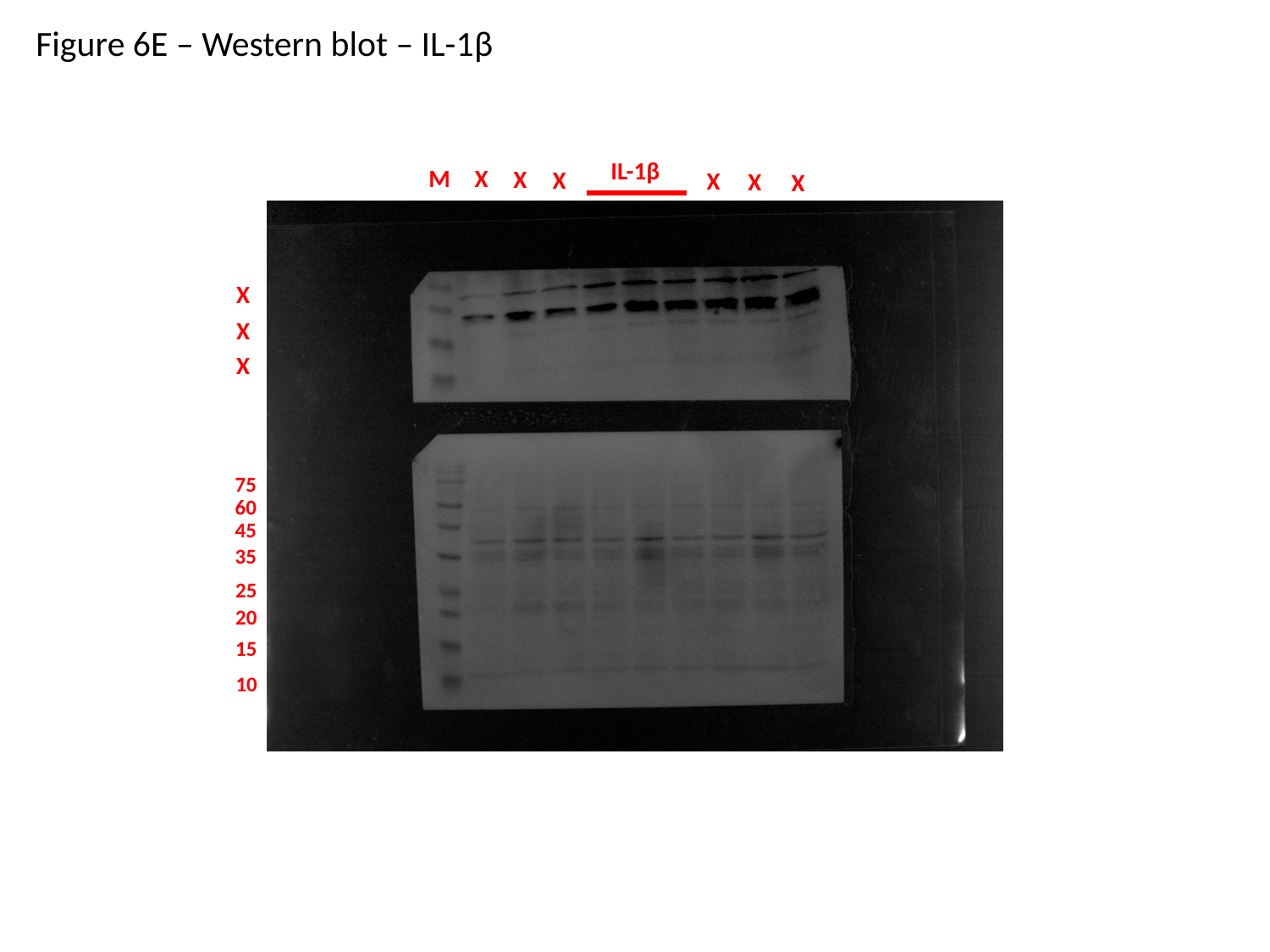

Figure 6E – Western blot – IL-1β
IL-1β
X
M
X
X
X
X
X
X
X
X
75
60
45
35
25
20
15
10

## Slide 8
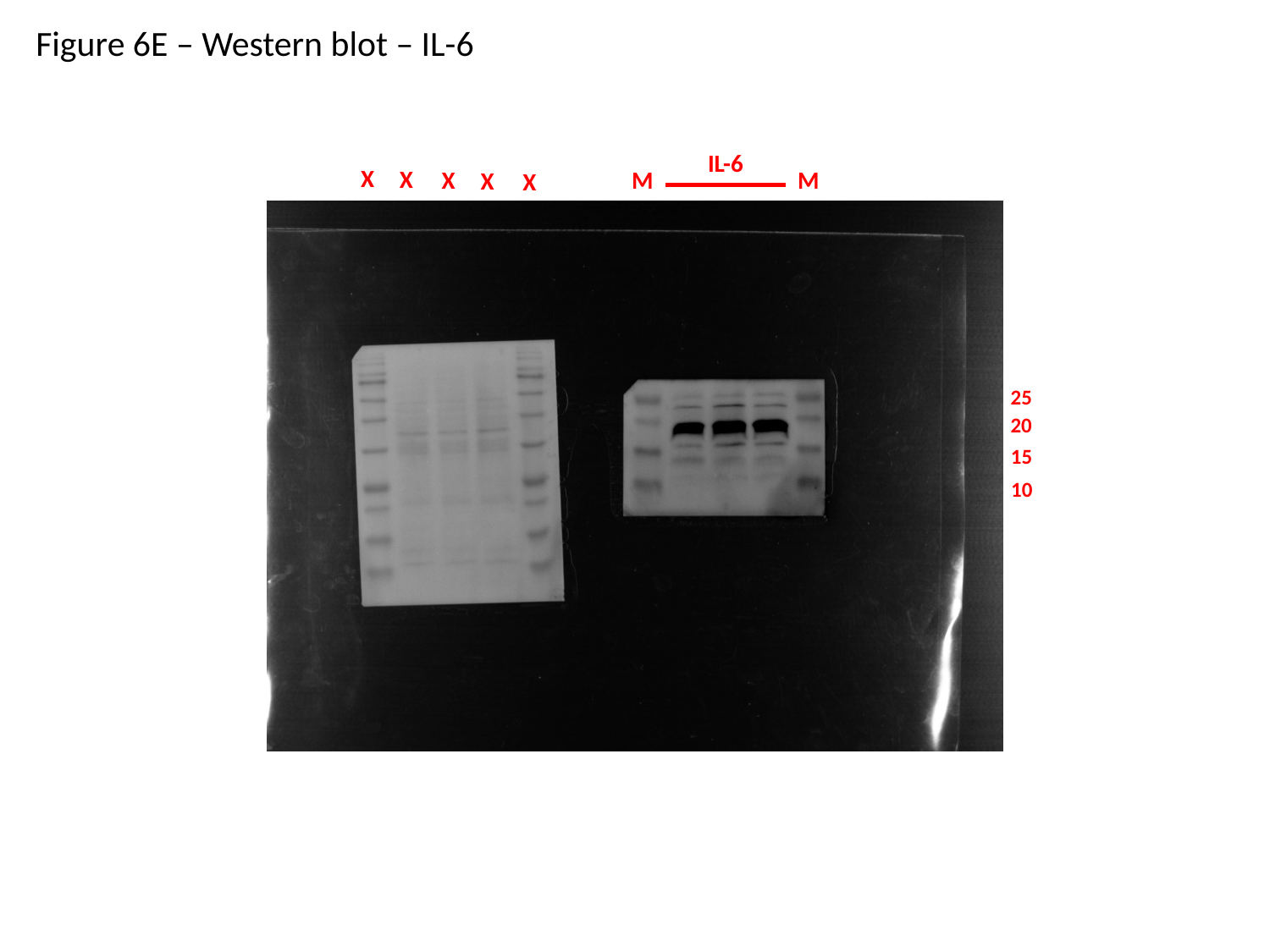

Figure 6E – Western blot – IL-6
IL-6
X
X
X
M
M
X
X
25
20
15
10

## Slide 9
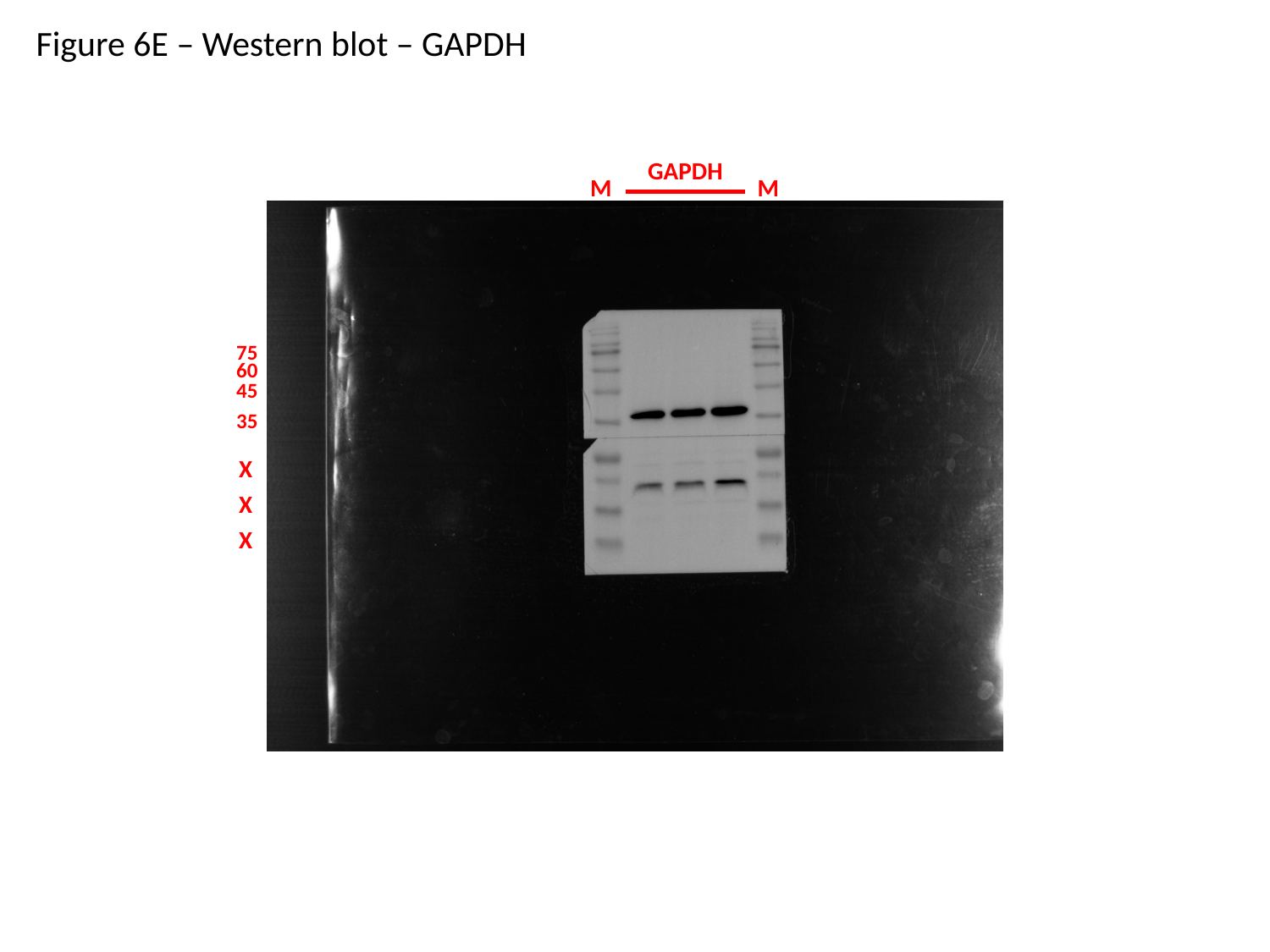

Figure 6E – Western blot – GAPDH
GAPDH
M
M
75
60
45
35
X
X
X
